# The Association Between Subclinical Thyroid Dysfunction and Recurrence of Atrial Fibrillation After Catheter Ablation

**DOI:** 10.3389/fcvm.2022.902411

**Published:** 2022-06-03

**Authors:** Rui-bin Li, Xiao-hong Yang, Ji-dong Zhang, Dong Wang, Xiao-ran Cui, Long Bai, Lei Zhao, Wei Cui

**Affiliations:** Department of Cardiology, The Second Hospital of Hebei Medical University, Shijiazhuang, China

**Keywords:** atrial fibrillation, radiofrequency catheter ablation, recurrence, subclinical thyroid dysfunction, subclinical hyperthyroidism, subclinical hypothyroidism

## Abstract

**Objective:**

The aim of this study was to evaluate the association between subclinical thyroid dysfunction and the recurrence of atrial fibrillation (AF) after radiofrequency catheter ablation (RFCA).

**Methods:**

We examined the association between subclinical thyroid dysfunction and the recurrence of AF at a large university-affiliated cardiac arrhythmia center in China. Data were collected from consecutive patients who underwent RFCA for AF, excluding those with a history of hypothyroidism, hyperthyroidism, or ongoing medical treatment for hypothyroidism or hyperthyroidism, biochemically defined overt thyroid disease, and long-term use of amiodarone before admission. The primary end point was the recurrence of AF in a time-to-event analysis. We compared outcomes in patients who had subclinical hyperthyroidism or hypothyroidism with those who had euthyroid state, using a multivariable Cox model with inverse probability weighting and propensity score matching.

**Results:**

In all, 93 patients were excluded from 435 consecutive patients who underwent RFCA for AF. Of the remaining 342 patients for the analysis, the prevalence of subclinical hyperthyroidism and subclinical hypothyroidism were 26 (7.6%) and 41 (12.0%), respectively; during a median follow-up of 489 days, 91 patients (26.6%) developed a primary end point event. In the main analysis of the multivariable Cox model, only subclinical hyperthyroidism [hazard ratio: 3.07, 95% confidence interval (CI): 1.54–6.14] was associated with an increased risk of end point event after adjusting for potential confounders. However, the association between subclinical hypothyroidism and the end point event was not significant (hazard ratio: 0.66, 95% CI: 0.31–1.43). Results were consistent either in multiple sensitivity analyses or across all subgroups of analysis. Compared with individuals with free triiodothyronine (fT3) in the lowest quintile, those with fT3 in the highest quintile had an HR of 2.23 (95% CI: 1.16–4.28) for recurrence of AF. With the increase of thyroid-stimulating hormone (TSH), a reduction in the risk of recurrence of AF was detected in the adjusted model, and the hazard ratio (HR) per standard deviation (SD) increase was 0.82 (95% CI: 0.68–0.98).

**Conclusion:**

In this retrospective cohort study involving patients who underwent RFCA for AF, patients with subclinical hyperthyroidism were associated with a markedly higher prevalence of recurrence of AF, whereas patients with subclinical hypothyroidism had a similar recurrence rate of AF compared to those with the euthyroid state.

## Introduction

Atrial fibrillation (AF) is known as the most common cardiac arrhythmia in adults worldwide. Approximately 1 in 3 individuals will develop this arrhythmia in a lifetime. In addition, AF leads to significant disability and mortality, which in consequence portends a heavy burden on patients and social health (1).

Rhythm control therapies for AF include antiarrhythmic drugs (AADs) that are often ineffective in preventing recurrence. Catheter ablation, which has been the first-line treatment for symptomatic AF (1), could not prevent the recurrence of AF either (2), probably because of the persistence of the modifiable risk factors (1) and arrhythmogenic substrate (3). Furthermore, early intervention of modifiable risk factors has been recommended to improve catheter ablation in AF (1).

Subclinical thyroid dysfunction, which includes subclinical hyperthyroidism and hypothyroidism [defined as decreased or elevated thyroid-stimulating hormone (TSH), respectively, with free triiodothyronine (fT3) and free thyroxine (fT4) in the normal range] (4), is prevalent, with 1–10% of the adults being affected by subclinical hyperthyroidism (5–7) and up to 20% by subclinical hypothyroidism (4, 8). Additionally, subclinical thyroid dysfunction is also a common condition among patients with AF (9). The association between subclinical hyperthyroidism and the incidence of AF has been well established (10–14). Many studies have also found an association between subclinical hypothyroidism and increased insulin resistance, dyslipidemia, hypertension (4), left ventricular dysfunction (15), increased prevalence of aortic atherosclerosis, and myocardial infarction (16), all of which predispose to AF.

Given the association between subclinical thyroid dysfunction and AF occurrence and the high prevalence of subclinical thyroid dysfunction among patients with AF, subclinical thyroid dysfunction may be associated with the outcome of catheter ablation for AF too. However, very little evidence in this regard has been established. Therefore, we conducted this retrospective cohort study to elucidate the association between subclinical thyroid dysfunction and the recurrence of AF after RFCA, considering the euthyroid state as a reference.

## Materials and Methods

This was a retrospective cohort study conducted at the Second Hospital of Hebei Medical University, a large university-affiliated cardiac arrhythmia center in China. The study was approved by the Research Ethics Committee of the Second Hospital of Hebei Medical University. All patients gave written informed consent before the AF ablation procedure.

### Data Source

The data was obtained from an electronic data warehouse (http://hbeyxn.edc-china.com.cn/login.jsp) of AF undergoing RFCA in our hospital. This disease-specific warehouse was established on 1 January 2017, and information from all consecutive patients with AF undergoing RFCA in our department was included in this database.

In the database, clinical and demographic characteristics of all patients were recorded, including sex, age, height, weight, smoking and drinking status, medical history, patient symptoms, laboratory tests, medication use, and the operation information. AADs were used for 3 months after AF ablation if there were no contraindications, and AADs were discontinued after 3 months if there was no recurrence of atrial tachyarrhythmia. Follow-up appointments in our outpatient electrophysiological (EP) clinic were scheduled at 3, 6, and 12 months for the first year and 6 months thereafter; unscheduled visits could be performed at any time if necessary. At each visit, a detailed medical and physical examination, 12-lead electrocardiogram, 24-h Holter, and cardiac ultrasound were performed. All baseline and subsequent follow-up visit measurements were entered into the AF database by an experienced investigator.

### Study Population

Consecutive patients who received an initial RFCA for drug-refractory paroxysmal or persistent AF between 1 January 2017 and 1 June 2021 at our department were included. Follow-up continued through 1 December 2021, by which time all the participants had completed at least 6 months of follow-up. The diagnosis of AF was based on an established practice guideline (1). Exclusion criteria were known thyroid disease, which was defined as a previous history of hypothyroidism, hyperthyroidism, or ongoing medical treatment for hypothyroidism or hyperthyroidism, biochemically defined overt thyroid disease and long-term use of amiodarone for more than 3 months before admission.

### AF Ablation Procedures

Treatment decisions (medical therapy or RFCA) were mainly based on practice guidelines at that time. RFCA was performed by two specialized physicians, including Ji-dong Zhang and Rui-bin Li, with more than 1,000 and 500 cases of experience in AF RFCA, respectively. The procedure was performed under local anesthesia and mild conscious sedation. The CARTO3 system (Biosense Webster, Diamond Bar, CA, USA) was used for three-dimensional electroanatomic left atrium (LA) reconstruction; ablation was performed with a 3.5-mm-tip irrigated catheter (TheromoCool SmartTouchTM, Biosense Webster, Diamond Bar, CA, USA). All patients achieved pulmonary vein isolation (PVI), and we checked the completeness of the two circular lesions in all patients with a decapolar circular catheter (LassoTM) or a multipolar mapping catheter with a 2-6-2 interelectrode spacing catheter (PentaRay, Biosense Webster, Diamond Bar, CA, USA). Decisions on whether to perform additional ablations (cavotricuspid isthmus, superior vena cava, arrhythmogenic substrate modification, or LA linear ablation) were left to the discretion of the operator. In patients who needed linear atrial lesions, the bidirectional conduction block across the lesions would be assessed by differential pacing maneuvers.

### Exposure Measurement

All blood samples were drawn in the morning in the fasting state after hospitalization. Electrochemiluminescence immunoassays for TSH, fT4, and fT3 were performed using the Elecsys detection method (Cobas Elecsys, Roche Diagnostics GmbH). The thyroid function can be classified as the euthyroid state (0.45 mIU/L ≤ TSH ≤ 4.5 mIU/L), subclinical hyperthyroidism (TSH < 0.45 mIU/L with fT4 and fT3 within the reference range), and subclinical hypothyroidism (TSH 4.51–19.99 mIU/L with fT4 level within the reference range) (17, 18). All measurements were performed by laboratory staff unaware of the patients' clinical characteristics.

### Outcome

The end point event was a recurrence of AF after RFCA. Recurrence was defined as documented AF, atrial flutter (AFL), or atrial tachycardia (AT) lasting for more than 30 s after the 3-month blanking period.

### Covariates Assessed

We obtained the following covariates, which were associated with AF for each patient from the clinical electronic data warehouse: age, sex, body mass index (BMI), patient-reported smoking status and alcohol use, AF pattern (paroxysmal AF and persistent AF) and AF duration (since the earliest evidence of AF), CHA2DS2-VASc score, duration of hospital stay, the first recorded inpatient laboratory tests (hemoglobin, fasting glucose, creatinine, and total cholesterol), echocardiogram results (left atrial diameter and left ventricular ejection fraction), past diagnoses (coronary artery disease, hypertension, diabetes, stroke, and heart failure), medication administration at baseline (diuretics, statin, angiotensin-converting enzyme inhibitors/angiotensin-receptor blockers, and β-blocker, calcium channel blockers), and procedure parameters (procedure time, radiofrequency power, and ablation strategy).

The BMI was defined as the weight in kilograms divided by the square of the height in meters. All blood samples were drawn in the morning in the fasting state. Past diagnoses were defined based on the clinical history or specific treatments. Medication information was obtained from preadmission medication lists or treatment plans that were followed from the day of admission. In the AF pattern, persistent and long-standing persistent AFs were collectively classified as persistent AF. The ablation strategy included PVI and PVI Plus (cavotricuspid isthmus, superior vena cava, arrhythmogenic substrate modification, or LA linear ablation beyond PVI).

### Statistical Analysis

Data were given as means with standard deviation (SD) for normally distributed variables, as medians and interquartile range (IQR) for skewed data, and as frequencies (percentage) for categorical variables. Normality of continuous variables was tested by the Shapiro-Wilk test; variance homogeneity was verified by the Bartlett test. Differences between groups were assessed using the chi-squared test or Fisher's exact test for categorical variables, and the Student's *t*-test or the Mann–Whitney U test for continuous variables.

Multivariate survival analyses were performed using the Cox proportional hazards model to estimate the association between subclinical thyroid dysfunction and the end point of recurrence of AF. Proportional hazard assumptions were checked using Schoenfeld residuals, and no violations were found. According to the recommendation of the STROBE statement (19), we simultaneously showed the results of unadjusted and adjusted analyses. All the analyses were first adjusted for age and sex (model 1) and subsequently using the lowest Akaike Information Criterion (AIC) for stepwise backward/forward model selection (model 2) (20). Furthermore, a fully adjusted multivariate Cox regression model with the euthyroid state as reference included demographic factors, clinical information, laboratory and echocardiogram results, medications, and procedure parameters (model 3). The survival and recurrence curves were estimated using the Kaplan–Meier method and compared using the Log-rank test.

To help account for the non-randomized distribution of the covariates, we conducted a second analysis using three propensity score (PS) methods. In the PS analysis, the euthyroid state was taken as the control group; PS was calculated in patients with subclinical hyperthyroidism and subclinical hypothyroidism, respectively. Inverse probability treatment weighting (IPTW) by PS was applied first. We fitted a logistic regression model of subclinical hyperthyroidism that regressed the same covariates as the Cox regression model and obtained the predicted probability of subclinical hyperthyroidism compared to euthyroid. We used the predicted probabilities to calculate the stabilized IPW weight. And then, we did the same calculation for subclinical hypothyroidism. Supplementary Materials provided more detailed methods for the propensity score model and IPTW. Propensity score matching (PSM) was then conducted by nearest-neighbor matching without replacement with an algorithm of 1:2 matching. PSM was conducted separately in patients with subclinical hyperthyroidism and subclinical hypothyroidism like IPTW. A standardized mean difference (SMD) was assessed to evaluate the balancing of covariates between matching groups (21). The results of the balancing test are shown in the Supplementary Materials. Finally, PS was further adjusted as an additional covariate. The Cox models and Kaplan–Meier curves based on IPTW and PSM were reported.

Stratified analyses were performed to estimate the associations of subclinical thyroid dysfunction and the end point event according to gender (male and female) and age (<65 and ≥65 years) because TSH distribution differed significantly by age and sex (18). The cutoff for age was determined to avoid inappropriately small participant numbers within individual subgroups. And we further conducted subgroup analyses on different AF patterns (paroxysmal AF and persistent AF). The likelihood ratio test was used to calculate *P*-values to test for interaction in the subgroup analyses.

Missing values were interpolated by multivariate imputation by chained equations based on random forests (22). Using multiple imputations, we generated five complete datasets, results based on the first imputed dataset were presented, and the other results were similar, as shown in the Supplementary Materials.

Multivariate Cox proportional hazards regression models were used to estimate the hazard ratio (HR) of recurrence of AF for TSH, fT4, and fT3 concentrations, and similar analyses were conducted for TSH, fT4, and fT3 quintiles. Both the unadjusted and the fully adjusted models were reported. Due to the skewness of the distribution, TSH, fT4, and fT3 were modeled with Log transformation, and SD units centered on the mean were reported to make TSH, fT3, and fT4 values comparable and interpretable across studies. At the same time, the untransformed outcomes are presented in the Supplementary Materials. We used restricted cubic spline curves based on Cox proportional hazards models with 3 knots at the 10th, 50th, and 90th percentiles of TSH, fT4, and fT3 to flexibly model the association of TSH, fT4, and fT3 with the recurrence of AF. Spline regression analyses were adjusted for potential confounders. In the spline models, the potential non-linearity was evaluated using the likelihood ratio test comparing the models, namely, linear and non-linear (23).

The statistical analyses were performed using the R software, version 4.1.2 (R Project for Statistical Computing). *P*-values < 0.05 were considered to indicate a significant difference.

## Results

### Characteristics of the Cohort

**Figure 1:**
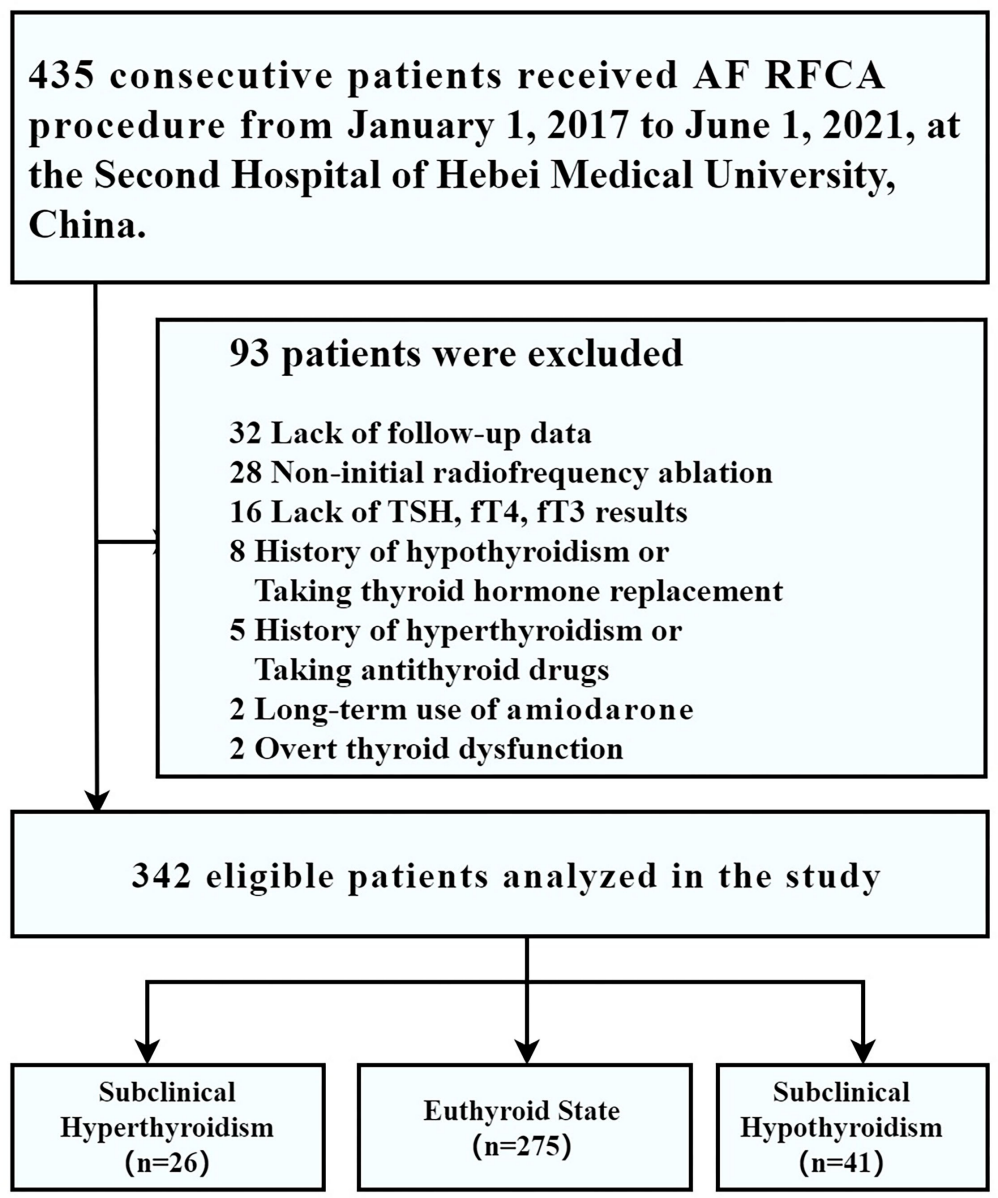
Study cohort.

From 1 January 2017 to 1 June 2021, 435 consecutive patients who underwent RFCA for drug-refractory symptomatic AF were enrolled in our disease-specific database. Among them, 32 patients were excluded owing to lack of follow-up data; 28 patients were excluded because of non-initial radiofrequency ablation; 16 were excluded for lack of TSH, fT4, and fT3 results; 15 patients were excluded because of a history of hypothyroidism (*n* = 8) or a history of hyperthyroidism (*n* = 5), current overt thyroid dysfunction (*n* = 2); 2 patients were excluded for the long-term use of amiodarone, thus leaving 342 patients for the analysis (subclinical hyperthyroidism, *n* = 26; euthyroid state, *n* = 275; subclinical hypothyroidism, *n* = 41) (Figure 1).

The prevalence of subclinical hyperthyroidism and hypothyroidism in our study were 26 (7.6%) and 41 (12.0%), respectively. The median age was 63 years at inclusion, 41.2% were women, the median AF duration was 12.37 months, 65.2% were paroxysmal AF, the median procedure time was 120 min, and 11.4% of patients underwent additional ablation beyond PVI. More detailed characteristics of the study population are shown in Table 1.

**Table 1:** Baseline characteristics of included participants stratified by thyroid function.

**Characteristic**	**Overall** **(*n* = 342)**	**Subclinical hyperthyroidism** **(*n* = 26)**	**Euthyroid state** **(*n* = 275)**	**Subclinical hypothyroidism** **(*n* = 41)**
**Demographic data**				
Age (year)	63.00 (56.00, 69.00)	65.50 (55.50, 71.25)	63.00 (56.00, 69.00)	63.00 (57.00, 68.00)
Gender (Female)	141 (41.2)	8 (30.8)	114 (41.5)	19 (46.3)
BMI (kg/m2)	24.49 (23.15, 27.06)	23.94 (23.00, 25.30)	24.80 (23.42, 27.05)	24.44 (22.72, 27.68)
Current smoking	53 (15.5)	3 (11.5)	46 (16.7)	4 (9.8)
Current drinking	46 (13.5)	3 (11.5)	39 (14.2)	4 (9.8)
Paroxysmal AF	223 (65.2)	14 (53.8)	181 (65.8)	28 (68.3)
CHA2DS2-VASc score	2.00 (1.00, 3.00)	1.50 (1.00, 3.00)	2.00 (1.00, 3.00)	2.00 (1.00, 3.00)
AF duration (months)	12.37 (1.03, 39.57)	24.33 (1.02, 62.72)	12.63 (1.20, 40.07)	6.07 (0.95, 24.35)
**Echocardiogram**				
LAD^*^ (mm)	37.14 (5.86)	37.93 (7.19)	37.13 (5.69)	36.60 (6.29)
LVEF (%)	65.40 (60.35, 68.65)	60.40 (56.70, 67.20)	65.90 (60.75, 69.20)	64.65 (60.92, 66.70)
**Initial laboratory tests**				
HGB(g/L)	140.00 (130.00, 149.00)	140.00 (127.75, 149.00)	140.00 (131.00, 149.00)	137.50 (129.75, 148.00)
Fasting glucose (mmol/L)	5.18 (4.76, 5.78)	5.12 (4.51, 6.24)	5.20 (4.79, 5.74)	4.92 (4.48, 5.56)
Creatinine (umol/L)	72.00 (61.00, 82.85)	80.00 (75.25, 85.25)	71.00 (61.00, 83.00)	72.00 (60.75, 80.50)
Total cholesterol (mmol/L)	3.99 (3.42, 4.59)	3.74 (3.03, 4.34)	4.09 (3.55, 4.60)	3.80 (3.22, 4.24)
**Past diagnoses**				
CAD	59 (17.3)	4 (15.4)	46 (16.7)	9 (22.0)
Hypertension	186 (54.4)	14 (53.8)	154 (56.0)	18 (43.9)
Diabetes	40 (11.7)	4 (15.4)	30 (10.9)	6 (14.6)
Stroke	39 (11.4)	6 (23.1)	29 (10.5)	4 (9.8)
HF	36 (10.5)	4 (15.4)	27 (9.8)	5 (12.2)
**Medications at baseline**				
Diuretics	74 (21.6)	7 (26.9)	61 (22.2)	6 (14.6)
Statin	117 (34.2)	9 (34.6)	89 (32.4)	19 (46.3)
ACEI/ARB	78 (22.8)	4 (15.4)	64 (23.3)	10 (24.4)
β-blockers	127 (37.1)	11 (42.3)	99 (36.0)	17 (41.5)
CCB	50 (14.6)	1 (3.8)	43 (15.6)	6 (14.6)
**Procedure parameters**				
Procedure time (min)	120.00 (100.00, 130.00)	120.00 (120.00, 130.00)	120.00 (100.00, 130.00)	120.00 (90.00, 132.00)
RF power (W)	35.00 (30.00, 40.00)	35.00 (33.75, 40.00)	40.00 (30.00, 40.00)	35.00 (30.00, 40.00)
Duration of hospital stay (Days)	7.00 (6.00, 9.00)	7.00 (6.00, 8.00)	7.00 (6.00, 9.00)	8.00 (6.00, 10.00)
Ablation strategy (PVI Plus)	39 (11.4)	3 (11.5)	27 (9.8)	9 (22.0)
**Thyroid condition**				
fT3 (pmol/L)	4.86 (4.43, 5.44)	5.62 (4.64, 6.45)	4.86 (4.46, 5.32)	4.60 (4.21, 5.51)
fT4 (pmol/L)	15.66 (14.21, 17.40)	17.90 (14.63, 22.52)	15.62 (14.26, 17.30)	15.37 (14.00, 16.70)
TSH (mIU/L)	1.93 (1.15, 3.00)	0.07 (0.02, 0.39)	1.85 (1.22, 2.58)	6.04 (5.30, 8.13)

*AF, atrial fibrillation; LAD, left atrium diameter; LVEF, left ventricular ejection fraction; BMI, body mass index; HGB, hemoglobin; CAD, coronary artery disease; HF, heart failure; ACEI, angiotensin-converting enzyme inhibitors; ARB, angiotensin-receptor blockers; CCB, calcium channel blockers; PVI Plus, cavotricuspid isthmus, superior vena cava, arrhythmogenic substrate modification or LA linear ablation beyond pulmonary vein isolation; RF power, radiofrequency power; TSH, thyroid-stimulating hormone; fT4, free thyroxine; fT3, free triiodothyronine*.

* *Data is given as mean (SD); the other data are given as medians (interquartile range) or frequencies (percentages). Data on the LAD and LVEF level are missing for 87 patients, RF power for 71, on the CHA2DS2-VASc score level for 62, on the AF duration level for 35, on the fasting glucose for 28, on the total cholesterol for 16, on the procedure time for 14, creatinine for 8, HGB for 6, and BMI for 5*.

The odds ratios (with 95% CI) of all the variables included in the logistic PS models are shown in Supplementary Table 1. The C-statistics for the PS models were 0.792 and 0.790, respectively (Supplementary Figure 1). Compared to the euthyroid state, the distribution of the PS for subclinical hyperthyroidism and subclinical hypothyroidism before and after PSM is shown in Supplementary Figure 2.

In the subclinical hyperthyroidism and euthyroid matched analytic samples, 52 euthyroid patients and 26 subclinical hyperthyroidism patients were matched. The differences between variables were attenuated in the matched samples vs. unmatched samples (Supplementary Table 2; Supplementary Figure 3). The subclinical hypothyroidism and euthyroid-matched samples showed similar results (Supplementary Table 2; Supplementary Figure 3). Based on the PS models, stabilized IPTW was applied, and the summaries of the data after IPTW are shown in Supplementary Table 3 and Supplementary Figure 4.

### Study End Point

Over a median of 489 days (maximum 1,430 days) of follow-up of the 342 patients in the cohort, the end point event of recurrence of AF developed in 91 patients (26.6%) (subclinical hyperthyroidism, 13/26, 50.0%; euthyroid state, 69/275, 25.1%; subclinical hypothyroidism, 9/41, 22.0%).

**Figure 2:**
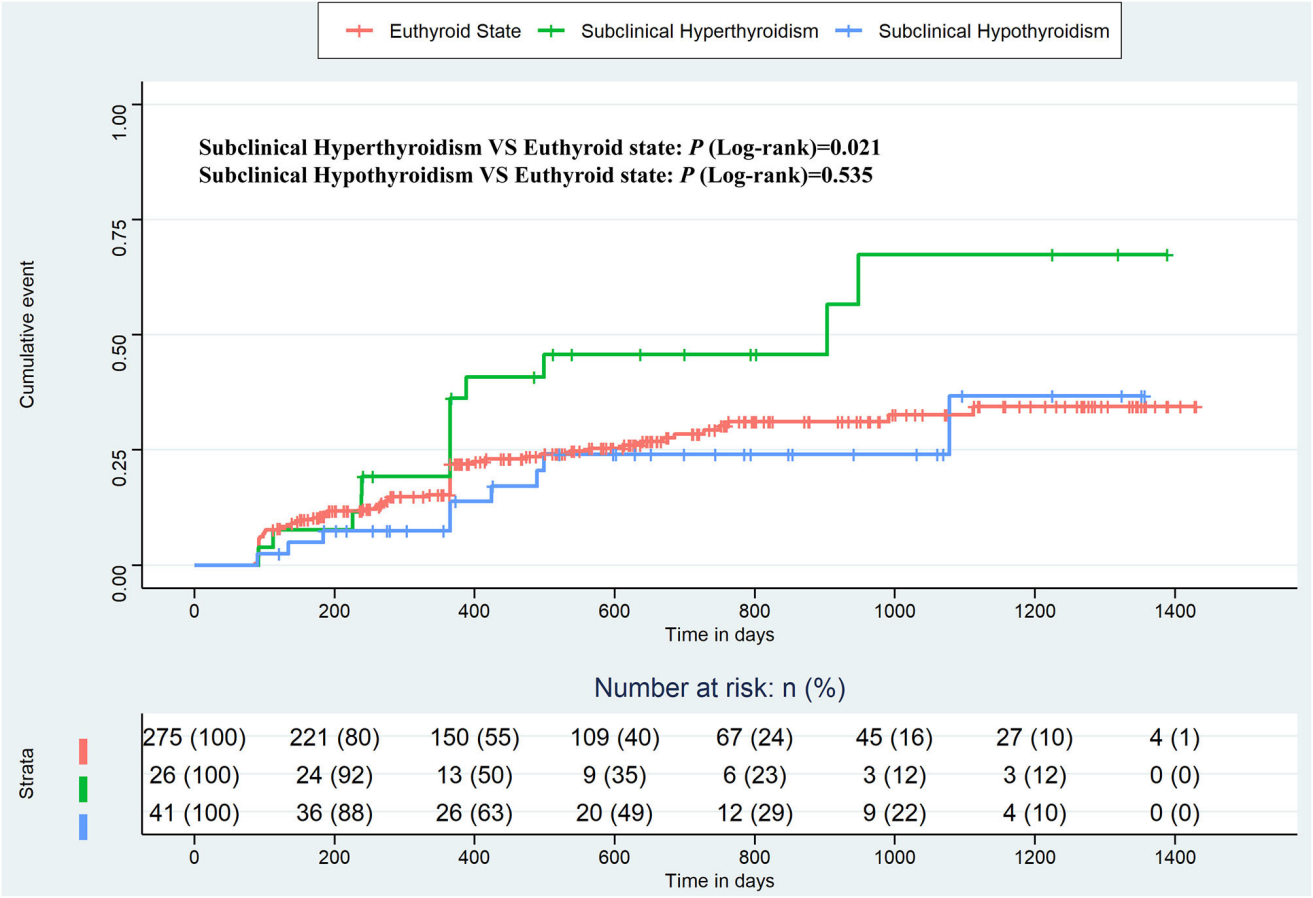
Kaplan–Meier plot estimates of the rate of recurrence of atrial fibrillation according to thyroid function, considering the euthyroid state as a reference.

**Figure 3:**
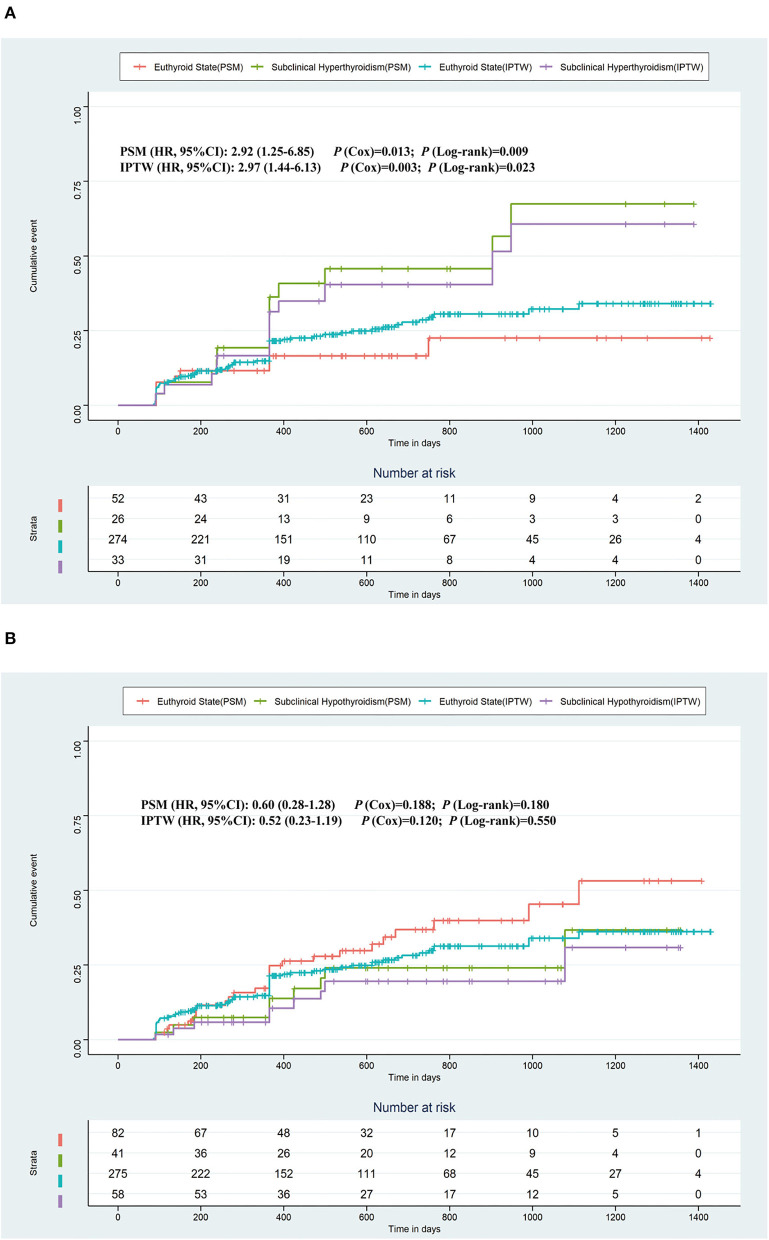
Kaplan–Meier plot estimates of the rate of recurrence of atrial fibrillation based on PSM and IPTW. **(A)** Kaplan–Meier plot estimates of the rate of recurrence of atrial fibrillation in the populations who were with subclinical hyperthyroidism or the euthyroid state. **(B)** Kaplan–Meier plot estimates of the rate of recurrence of atrial fibrillation in the populations who were with subclinical hypothyroidism or the euthyroid state. PSM, propensity score matching; IPTW, inverse probability treatment weighting.

Figure 2 shows the Kaplan–Meier estimates of the recurrence rate of AF during follow-up, differentiating between patients with subclinical hyperthyroidism and hypothyroidism, compared to patients with a euthyroid state. The cumulative event was higher in patients with subclinical hyperthyroidism than in patients with the euthyroid states (Log-rank test, *p* = 0.021), but there was no significant difference in patients with subclinical hypothyroidism compared to patients with normal thyroid function (Log-rank test, *p* = 0.535). The Kaplan–Meier estimates based on PSM and IPTW are shown in Figure 3, all of which revealed that subclinical hyperthyroidism rather than subclinical hypothyroidism was associated with a significantly higher recurrence rate of AF.

To further determine the impact of subclinical thyroid dysfunction on the recurrence of AF, a stepwise Cox regression analysis adjusted for potential confounding factors was performed. In the crude analysis, patients who had subclinical hyperthyroidism were more likely to have an end point event than patients with the euthyroid state (HR: 1.98; 95% CI: 1.10–3.59), while patients with subclinical hypothyroidism were not (HR: 0.81; 95% CI: 0.40–1.61). In the primary multivariable analysis, only subclinical hyperthyroidism (HR: 2.12; 95% CI: 1.17–3.86) was associated with an increased risk of end point event compared with euthyroidism after adjusting for age and sex. The association between subclinical thyroid dysfunction and the recurrence of AF demonstrated similar results in models of different adjustment strategies and additional PS analyses (Table 2).

**Table 2:** Associations between subclinical thyroid dysfunction and the recurrence after radiofrequency catheter ablation for atrial fibrillation in the crude, multivariate, and PS analyses.

**Analyses**	**Recurrence**
**No. of events / No. at risk (%)**	
Subclinical hyperthyroidism	13/26 (50.0)
Euthyroid state	69/275 (25.1)
Subclinical hypothyroidism	9/41 (22.0)
**Crude analyses— HR (95% CI)**	
**Ref** **=** **Euthyroid state**	
Subclinical hyperthyroidism	1.98 (1.10–3.59)
Subclinical hypothyroidism	0.81 (0.40-1.61)
**Multivariable analyses— HR (95% CI)**	
Ref = Euthyroid State^*^	
**Model1**	
Subclinical hyperthyroidism	2.12 (1.17, 3.86)
Subclinical hypothyroidism	0.80 (0.40, 1.60)
**Model2**	
Subclinical hyperthyroidism	2.93 (1.56, 5.53)
Subclinical hypothyroidism	0.71 (0.34, 1.46)
**Model3**	
Subclinical hyperthyroidism	3.07 (1.54, 6.14)
Subclinical hypothyroidism	0.66 (0.31, 1.43)
**PS analyses— HR (95% CI)**	
**Ref= Euthyroid State**	
**With IPTW ^†^**	
Subclinical hyperthyroidism	2.97 (1.44, 6.13)
Subclinical hypothyroidism	0.52 (0.23, 1.19)
**With PSM ‡**	
Subclinical hyperthyroidism	2.92 (1.25, 6.85)
Subclinical hypothyroidism	0.60 (0.28, 1.28)
**Adjusted for PS§**	
Subclinical hyperthyroidism	3.40 (1.65, 6.97)
Subclinical hypothyroidism	0.62 (0.28, 1.35)

* *Model1 with additional adjustment for age and gender. Model2 with additional adjustment for age, current smoking status, duration of hospital stay, LAD, HGB, hypertension, stroke, HF, diuretics, ACEI/ARB, procedure time, and RF power using Akaike information criterion (AIC) for model selection. Model3 with additional adjustment for age, BMI, gender, current smoking and drinking status, AF pattern, CHA2DS2-VASc score, AF duration, echocardiogram information, past diagnoses, current medications, laboratory tests, and procedure parameters. The analysis includes all 342 patients*.

† *Adjust for the same covariates in model3 with IPTW according to the PS. The analysis includes all 342 patients*.

*^‡^Without additional adjustment because of the well balance of the covariates after PSM. The analysis includes 78 patients (26 in subclinical hyperthyroidism state and 52 in the euthyroid state) and 123 patients (41 in subclinical hypothyroidism state and 82 in the euthyroid state), respectively*.

*^§^Adjust for the same covariates in model3, with additional adjustment for the PS. The analysis includes all 342 patients. HR, hazard ratio; CI, confidence interval; PSM, propensity score matching; PS, propensity score; IPTW, inverse probability treatment weighting*.

**Figure 4:**
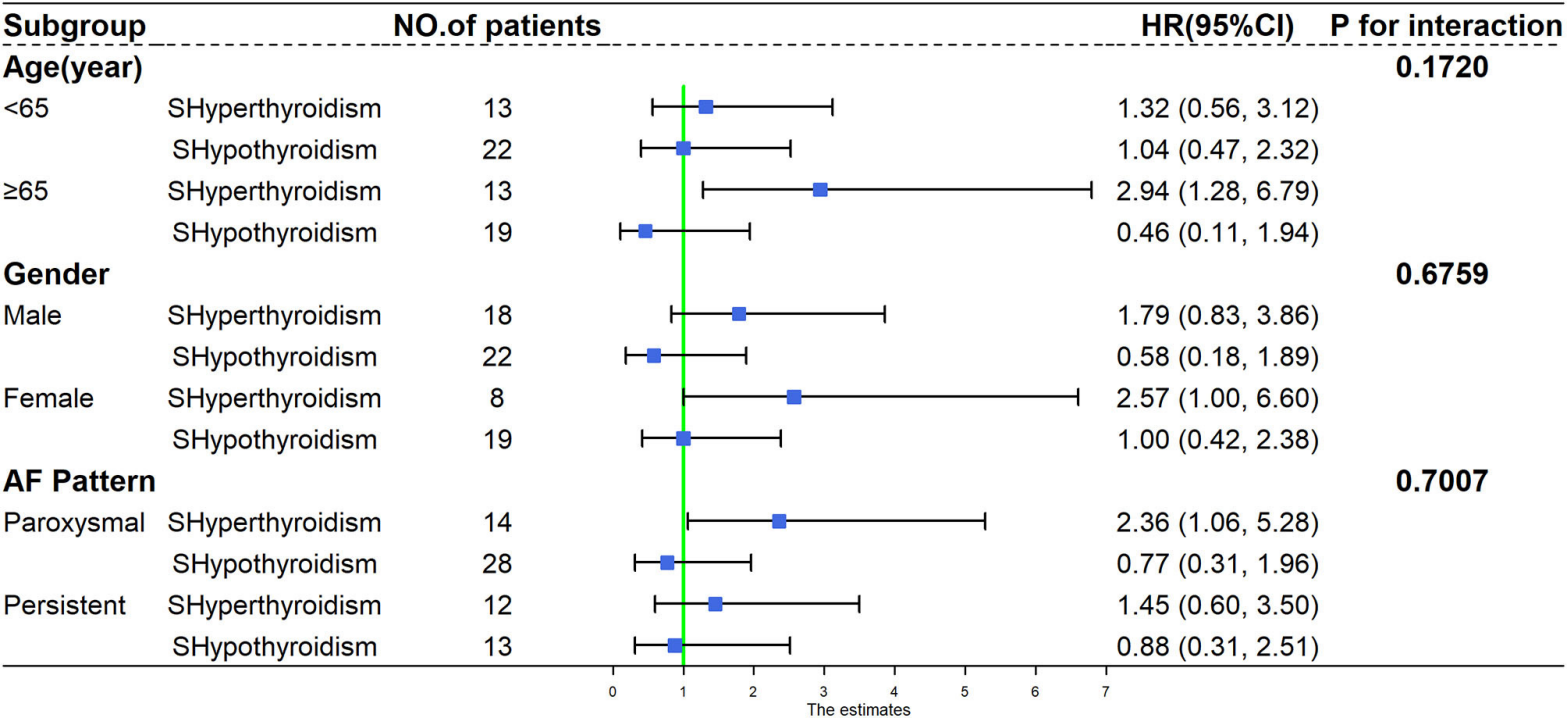
Subgroup analysis of recurrence of atrial fibrillation according to different age, gender, and atrial fibrillation pattern. HR, hazard ratio; CI, confidence interval; AF, atrial fibrillation; SHyperthyroidism, subclinical hyperthyroidism; SHypothyroidism, subclinical hypothyroidism.

We observed a similar trend of association between subclinical thyroid dysfunction and the recurrence of AF across all subgroups of analysis, and no significant interaction was found when analyses were stratified by age (<65 and ≥65 years), gender (male and female), and AF pattern (paroxysmal AF and persistent AF) (*P*-values for interaction were 0.1720, 0.6759, and 0.7007, respectively). The results of the stratified analysis are presented in Figure 4.

### Relationship Between TSH, fT3, fT4, and AF Recurrence

**Figure 5:**
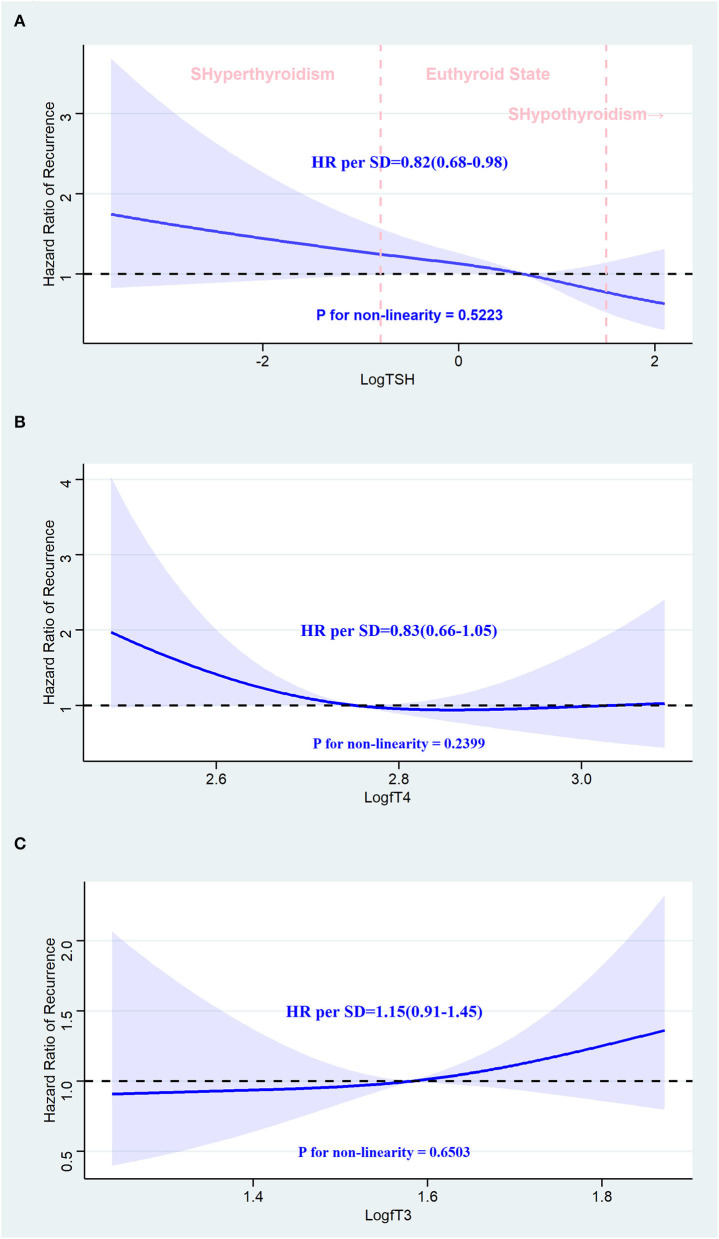
Association between thyroid hormone and recurrence of atrial fibrillation after radiofrequency catheter ablation using a restricted cubic spline regression model. **(A)** TSH and recurrence of AF after RFCA. **(B)** fT4 and recurrence of AF after RFCA. **(C)** fT3 and recurrence of AF after RFCA. Results are adjusted for age, BMI, gender, current smoking and drinking status, AF pattern, CHA2DS2-VASc score, AF duration, echocardiogram information, past diagnoses, current medications laboratory tests, and procedure parameters. The restricted cubic spline regression model is conducted with three knots at the 10th, 50th, and 90th percentiles of TSH, fT4, and fT3, and they are modeled with Log transformation. The blue ribbon represents the 95% CI for the spline model. Reference lines for no association are indicated by the dashed black lines at an HR of 1.0. HR, hazard ratio; CI, confidence interval; SD, standard deviation; SHyperthyroidism, subclinical hyperthyroidism; SHypothyroidism, subclinical hypothyroidism; AF, atrial fibrillation; RFCA, radiofrequency catheter ablation.

In Figure 5, we used restricted cubic splines to visualize the relationship between TSH, fT4, and fT3 and the recurrence of AF, and no statistically significant departure from linearity was detected (*p*-values for non-linearity were 0.5223, 0.2399, and 0.6503, respectively). Regarding the linear relationship between TSH and recurrence of AF, the plot showed a significant reduction in the risk of recurrence of AF with the increase in TSH, and the HR per SD increase was 0.82 (95% CI: 0.68–0.98) in the adjusted model. We also examined differences in recurrence rates across quintiles. Compared with individuals in the lowest quintile, the risk of AF recurrence slightly decreased with the second to fifth TSH quintiles, and the HRs were 1.06, 0.88, 0.94, and 0.70, respectively, but the trend was not significant (*p*-value for the trend is 0.291). Although TSH was negatively associated with the recurrence of AF in the crude model, this trend was not statistically significant (HR: 0.86; 95% CI: 0.72–1.01). When modeled as continuous measures, we detected associations between fT4 and fT3 and the recurrence of AF, and fT4 and fT3 were not associated with AF recurrence [fT4: crude model, HR: 0.84 (0.68, 1.04); adjusted model, HR: 0.83 (0.66, 1.05). fT3: crude model, HR: 1.15 (0.95, 1.40); adjusted model, HR: 1.15 (0.91, 1.45)]. When modeled as quintile categorical measures, compared with individuals with fT3 in the lowest quintile, those with fT3 in the highest quintiles had an HR of 2.23 (95% CI: 1.16–4.28) for recurrence of AF, while the HR was similar (HR: 2.13; 95% CI: 1.04–4.37) after adjusting for the potential confounders. Regarding the fT4 level, all of the models revealed that fT4 was not related to the recurrence of AF (Table 3).

**Table 3:** Recurrence of atrial fibrillation by fT3, fT4, and TSH as quintiles and fT3, fT4, and TSH as a continuous exposure at baseline.

**Variable**	**fT3**			**fT4**			**TSH**		
	**Events/N**	**Crude model** **(HR, 95%CI)**	**Adjusted model (HR, 95%CI)**	**Events/N**	**Crude model (HR, 95%CI)**	**Adjusted model** **(HR, 95%CI)**	**Events/N**	**Crude model** **(HR, 95%CI)**	**Adjusted model (HR, 95%CI)**
Per SD increase	91/342	1.15 (0.95, 1.40)	1.15 (0.91, 1.45)	91/342	0.84 (0.68, 1.04)	0.83 (0.66, 1.05)	91/342	0.86 (0.72, 1.01)	0.82 (0.68, 0.98)
**Quintiles**									
Q1	14/69	Ref	Ref	24/69	Ref	Ref	20/69	Ref	Ref
Q2	19/69	1.44 (0.72, 2.87)	1.80 (0.84, 3.85)	18/68	0.75 (0.41, 1.39)	0.57 (0.29, 1.12)	19/69	1.00 (0.53, 1.87)	1.06 (0.54, 2.11)
Q3	14/68	1.04 (0.49, 2.18)	0.85 (0.38, 1.88)	13/68	0.55 (0.28, 1.08)	0.58 (0.28, 1.18)	18/67	0.97 (0.51, 1.83)	0.88 (0.44, 1.76)
Q4	18/67	1.43(0.71, 2.88)	1.91(0.90, 4.05)	20/68	0.90(0.50, 1.63)	0.90(0.47, 1.76)	19/68	1.08(0.58, 2.03)	0.94(0.47, 1.89)
Q5	26/69	2.23 (1.16, 4.28)	2.13 (1.04, 4.37)	16/69	0.66 (0.35, 1.25)	0.62 (0.32, 1.22)	15/69	0.73 (0.37, 1.43)	0.70 (0.34, 1.41)
P for trend	–	0.024	0.055	–	0.342	0.463	–	0.485	0.291

*TSH, thyroid-stimulating hormone; fT4, free thyroxine; fT3, free triiodothyronine; AF, atrial fibrillation; CI, confidence interval; HR, hazard ratio; SD, standard deviation. Adjusted model: adjust for age, BMI, gender, current smoking and drinking status, AF pattern, CHA2DS2-VASc score, AF duration, echocardiogram information, past diagnoses, current medications, laboratory tests, and procedure parameters. TSH, fT4, and fT3 are modeled with Log transformation*.

## Discussion

To our knowledge, this is the first study focused on the association between the whole spectrum of subclinical thyroid dysfunction and the subsequent risk of recurrence of AF after RFCA. The major findings of our investigation are as follows: (1) patients with subclinical hyperthyroidism have a markedly higher prevalence of recurrence of AF after ablation procedures than those with euthyroid state, whereas the patients with subclinical hypothyroidism have the recurrence rate similar to those with euthyroid state and (2) high-normal fT3 level is associated with a significantly higher prevalence of recurrence of AF in the patients with all levels of subclinical thyroid dysfunction.

Many clinical studies have investigated the relationship between subclinical thyroid dysfunction and the new-onset of AF. The current evidence suggests an association between subclinical hyperthyroidism and AF. A population-based study of 5,860 people found that AF occurred in 9.5% of people with subclinical hyperthyroidism, compared with 4.7% in euthyroid individuals (10). In addition, in three prospective studies, a similar association between subclinical hyperthyroidism and AF was noted (11, 12, 14). In a retrospective cross-sectional study including 23,000 individuals with cardiovascular disease, the incidence of AF was similar in patients with subclinical hyperthyroidism (13%) and overt hyperthyroidism (14%), compared with 2% in patients with the euthyroid state (13). However, the role of subclinical hypothyroidism in atrial arrhythmogenesis has not been fully investigated, and a clear association has not been shown. A retrospective study of 586,460 participants found a protective effect of subclinical hypothyroidism on AF. On the contrary, subclinical hyperthyroidism was associated with the new onset of AF (24). In addition, no significant association between subclinical hypothyroidism and a 10-year risk of incident AF was detected in a community-based study (25). In two cohort studies of patients undergoing cardiac surgery, subclinical hypothyroidism was not associated with an increased risk of AF either (26, 27).

Until now, the evidence of the association between subclinical thyroid dysfunction and the recurrence of AF after RFCA has been sparse. Based on the evidence of subclinical thyroid dysfunction and the new onset of AF, it was reasonable to hypothesize that patients with subclinical hyperthyroidism rather than subclinical hypothyroidism were associated with the increased risk of recurrence of AF after RFCA. In accordance with that, we found an increased risk of recurrence of AF in patients with subclinical hyperthyroidism, while no significant association was detected between subclinical hypothyroidism and the recurrence of AF in this study.

Several studies have assessed the effects of thyroid hormone in the normal range on the recurrence of AF after RFCA. In 2010, it was first reported that a high-normal fT4 was related to the recurrence of AF after RFCA of paroxysmal AF (28). Subsequently, a large-scale study confirmed this association (29). Unfortunately, these studies did not include measurements of fT3. Wei reported that the association between the fT3 levels and the recurrence of AF followed a U-shape (30), which slightly differed from our findings. In a recent Chinese study, high-normal fT3 and fT4 levels were associated with a recurrence of AF after catheter ablation (31). Recently, a cohort study involving patients with overt hypothyroidism and subclinical hypothyroidism revealed that a high-normal TSH level might be an independent predictor of atrial tachyarrhythmia recurrence after catheter ablation of AF (32).

In this study, we focused on patients with subclinical thyroid dysfunction, defined as abnormal TSH with fT3 and fT4 in the normal range, in which the association between thyroid hormone and recurrence of AF after RFCA was little investigated. In our study, we detected an association between high-normal fT3 level and a higher risk for recurrence of AF, whereas fT4 was not. Furthermore, a lower concentration of TSH was negatively related to the recurrence of AF in our adjusted result; however, this result must be interpreted with caution because of the non-significant trend in the unadjusted model. And the difference in study population from the studies mentioned above might be the reason for the inconsistent results. Another difference from the previously published studies was that the participants in our study were defined based on comprehensive consideration of TSH, fT3, and fT4, which was in accordance with clinical practice for subclinical thyroid disease (4, 18). On the contrary, all of the aforementioned studies tried to discuss the relationship between thyroid hormones like TSH, fT3, and fT4 and the recurrence of AF separately. To our knowledge, we were the first to demonstrate an association between subclinical hyperthyroidism and a recurrence of AF after ablation procedures. None of the studies mentioned above were able to show such a relationship.

Due to the log-linear relationship between TSH and fT4, minor alterations in fT4 induced disproportionately larger changes in TSH (33). In addition, T3 has generally been considered to be the only biologically active form of thyroid hormone (34) and has had multiple effects on the cardiovascular system (35–37). As a result, TSH and T3 might play a more sensitive role in predicting arrhythmia recurrence after RFCA, which was supported by our findings and previous observational studies (12, 13, 24, 38–42). Higher thyroid hormone could shorten the action potential duration, decrease the speed of repolarization in pulmonary vein (PV) cardiomyocytes (43), and increase the frequency of atrial premature beats (44) and was correlated with more severe cardiac fibrosis, which facilitates the maintenance of multiple reentrant circuits in the heart (24, 45). These effects might explain the observation of an increased risk of recurrence of AF in patients with subclinical hyperthyroidism.

Regarding the strengths of our study, this is the first cohort investigating the whole spectrum of subclinical thyroid disease and the subsequent risk of recurrence of AF after RFCA. Our study has an adequate number of patients and a detailed evaluation of each participant. Moreover, we define subclinical thyroid dysfunction based on serum TSH, fT4, and fT3, which had not been implemented in previous studies. With the analytic approaches, we tried to minimize possible confounding factors in a variety of ways. We performed a series of analyses using several PS approaches and multivariate Cox regression analysis with different adjustment strategies. The findings are similar in the multiple analyses and different subgroups, and the consistency of the outcomes of these analyses is reassuring. The association between high level of fT3 and a higher risk of recurrence of AF further confirms our findings.

Our findings have great clinical implications. Subclinical hyperthyroidism behaves as an independent risk factor for the development of postoperative AF in patients undergoing RFCA, and maintenance of sinus rhythm may be improved by the treatment of modifiable risk factors (1). It has been reported that the prolonged atrial conduction time could be reversed by treatment of subclinical hyperthyroidism to restore biochemical euthyroidism (46). Furthermore, antithyroid therapy in patients with subclinical hyperthyroidism could reduce heart rate and improve supraventricular arrhythmias (47, 48). Our findings underline the importance of early detection and comprehensive management of thyroid function in patients who undergo RFCA for AF. However, future studies are needed to investigate whether antithyroid therapy and more aggressive postoperative adjuvant treatment in patients with subclinical hyperthyroidism could really provide a clinical benefit.

Our research has some limitations too. There are missing data for some variables. On the contrary, we used the multiple imputation method to account for missing data and minimize bias. The single-center design might hamper the generalization of our conclusions. We did not construct thyroid measurements as time-dependent covariates, so it was not possible to identify any changes in thyroid function over time. Finally, the recurrence of AF could have been asymptomatic, and monitoring using an implantable recorder could have revealed a higher recurrence rate.

## Conclusion

In this retrospective cohort study involving patients who underwent RFCA for AF, patients with subclinical hyperthyroidism had a significantly higher prevalence of recurrence of AF, whereas patients with subclinical hypothyroidism had a similar recurrence risk of AF compared to those in the euthyroid state.

## Data Availability Statement

The raw data supporting the conclusions of this article will be made available by the authors, without undue reservation.

## Ethics Statement

The studies involving human participants were reviewed and approved by the Research Ethics Committee of The Second Hospital of Hebei Medical University. Written informed consent for participation was not required for this study in accordance with the national legislation and the institutional requirements.

## Author Contributions

R-bL performed the statistical analyses, interpreted data, and drafted and revised the manuscript. X-hY, DW, LB, LZ, J-dZ, and X-rC acquired data. WC interpreted data, designed the study, revised the manuscript for important intellectual content, and approved the final version. All authors have read and approved the manuscript.

## Conflict of Interest

The authors declare that the research was conducted in the absence of any commercial or financial relationships that could be construed as a potential conflict of interest.

## Publisher's Note

All claims expressed in this article are solely those of the authors and do not necessarily represent those of their affiliated organizations, or those of the publisher, the editors and the reviewers. Any product that may be evaluated in this article, or claim that may be made by its manufacturer, is not guaranteed or endorsed by the publisher.

## Abbreviations

AF: atrial fibrillation
AADs: antiarrhythmic drugs
AFL: atrial flutter
AT: atrial tachycardia
ACEI: angiotensin-converting enzyme inhibitors
ARB: angiotensin-receptor blocker
AIC: Akaike Information Criterion
BMI: body mass index
CAD: coronary artery disease
CCB: calcium channel blocker
CI: confidence interval
EP: electrophysiological
fT4: free thyroxine
fT3: free triiodothyronine
HF: heart failure
HR: hazard ratio
HGB: hemoglobin
IPTW: inverse probability treatment weighting
LAD: left atrial diameter
LVEF: left ventricular ejection fraction
PVI: pulmonary vein isolation
PV: pulmonary vein
PSM: propensity score matching
RFCA: radiofrequency catheter ablation
SMD: standardized mean difference
TSH: thyroid-stimulating hormone
TC: total cholesterol
SD: standard deviation.
