# Supplementary material for: The Association Between Subclinical Thyroid Dysfunction and Recurrence of Atrial Fibrillation After Catheter Ablation
Source: Front Cardiovasc Med. 2022 Jun 3;9:902411. doi: 10.3389/fcvm.2022.902411 (PMC9203885; doi:10.3389/fcvm.2022.902411)
Supplement: Supplementary file 2 [file Data_Sheet_2.docx]

**Supplementary Appendix**

**Supplementary Methods** Propensity score model and inverse probability of treatment weighting

**Figure S1** Area Under Receiver Operating Characteristic (AUROC) curves for the propensity score models

**Figure S2** The distribution of the PS for subclinical hyperthyroidism and subclinical hypothyroidism before and after PSM.

**Figure S3** Standardized mean differences in the unmatched and matched sample

**Figure S4** Distribution of inverse probability score weights in the subclinical hyperthyroidism, subclinical hypothyroidism and euthyroid state groups

**Table S1** Odds ratios (95% CIs) of patients with subclinical hyperthyroidism or subclinical hypothyroidism for all variables included in the propensity score models

**Table S2** Baseline characteristics of the participants after PSM

**Table S3** Baseline characteristics of the participants after IPTW

**Table S4** Recurrence of atrial fibrillation by fT3, fT4, TSH quintiles and fT3, fT4, TSH as a continuous exposure based on the non-transformed data.

**Table S5** Hazard ratios (95% CIs) for the endpoint for all variables included as covariates in the Univariable and Multivariate Cox model

**Table S6** Associations between subclinical thyroid dysfunction and the recurrence rate after radiofrequency catheter ablation for atrial fibrillation in the Crude, Multivariate and PS Analyses based on the other imputed datasets.

**Supplementary Methods**

Propensity score model and inverse probability of treatment weighting

The propensity score-based inverse probability of treatment weighting (IPTW) was performed to minimise confounding. The method of IPTW based on the propensity score could generate a pseudo-population in which the distribution of baseline covariates was independent of treatment assignment (1).

The participants in our cohort were divided into three parts (Subclinical Hyperthyroidism, Euthyroid state, Subclinical Hypothyroidism). In the subclinical hyperthyroidism and Euthyroid state samples, only participants with Subclinical Hyperthyroidism and Euthyroid state were included in the analysis. We fitted a logistic regression model of Subclinical Hyperthyroidism (binary) to adjust other baseline covariates and obtained the predicted probability of Subclinical Hyperthyroidism. The variables included in the model were age, BMI, gender, current smoking and drinking status, AF pattern, CHA2DS2-VASc score, AF duration, echocardiogram information, past diagnoses, current medications, laboratory tests and procedure parameters. The predicted probabilities were used to calculate the stabilized IPTW. Each individual in the sample was weighted by the inverse probability of his or her treatment status, Wt=Pt/PS for the treatment group and Wc=(1-Pt)/(1-PS) for the control group (2, 3), where Pt was the proportion of accepting treatment factor in the whole population, and PS was the propensity score. IPTW with stabilization was accomplished by performing R code (4) below:

dat$iptw <-ifelse(dat$trt ==1,(mean(dat$ps))/dat$ps,(mean(1-dat$ps))/(1-dat$ps)).

In the subclinical hypothyroidism and Euthyroid state samples, only participants with Subclinical Hypothyroidism and Euthyroid state were included in the analysis, and the same calculation strategy was applied repeatedly.

REFERENCES

1. Austin PC. An Introduction to Propensity Score Methods for Reducing the Effects of Confounding in Observational Studies. Multivariate Behav Res. (2011) 46:399-424. doi: 10.1080/00273171.2011.568786

2. Robins JM, Hernán MA, Brumback B. Marginal structural models and causal inference in epidemiology. Epidemiology. (2000) 11:550-60. doi: 10.1097/00001648-200009000-00011

3. Hernán MA, Brumback B, Robins JM. Marginal structural models to estimate the causal effect of zidovudine on the survival of HIV-positive men. Epidemiology. (2000) 11:561-70. doi: 10.1097/00001648-200009000-00012

4. Lee J, Little TD. A practical guide to propensity score analysis for applied clinical research. Behav Res Ther. (2017) 98:76-90. doi: 10.1016/j.brat.2017.01.005

Figure S1 Area Under Receiver Operating Characteristic (AUROC) curves for the propensity score models


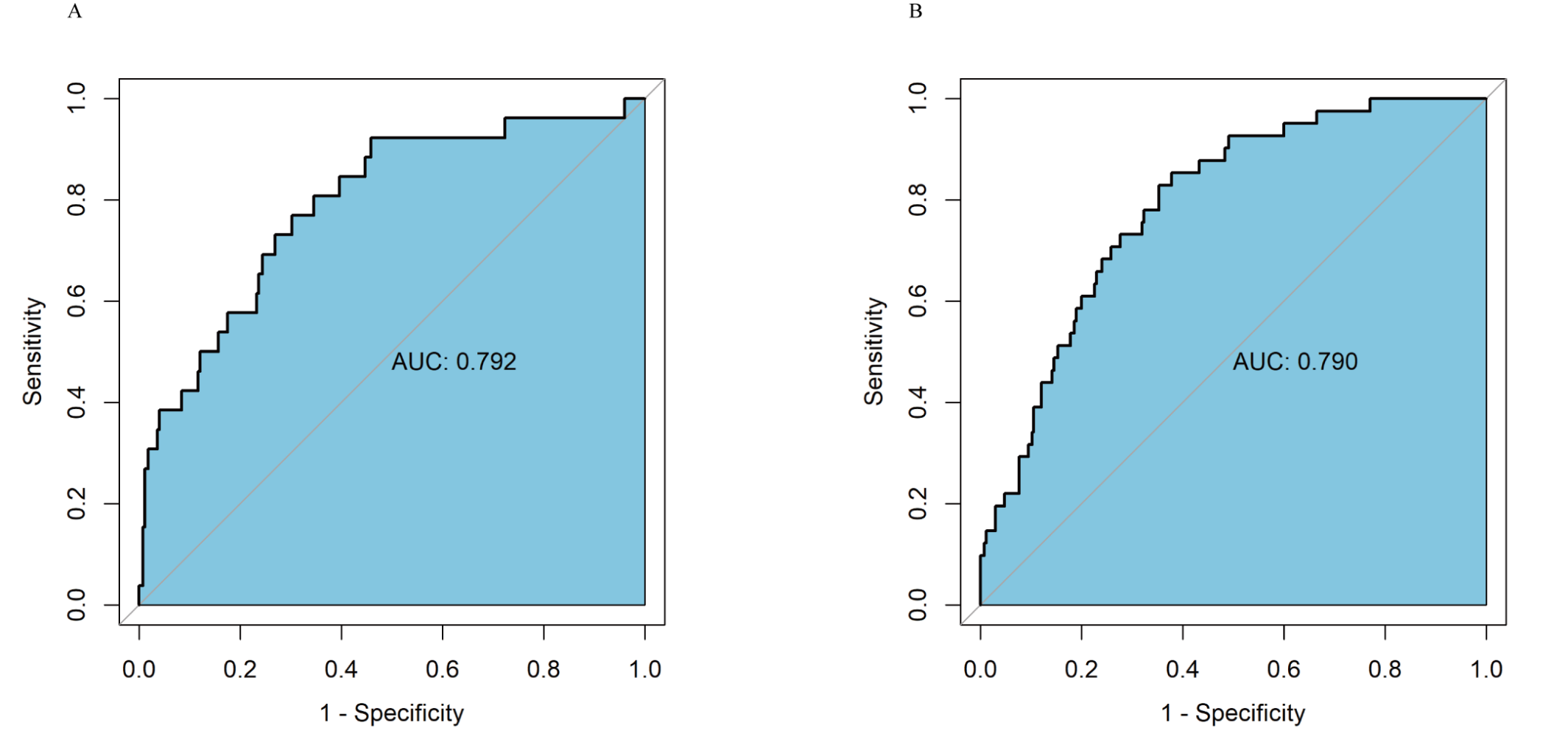


A: AUC of the propensity score model in the populations who were with subclinical hyperthyroidism or euthyroid state. B: AUC of the propensity score model in the populations who were with subclinical hypothyroidism or euthyroid state.

Figure S2 The distribution of the PS for subclinical hyperthyroidism and subclinical hypothyroidism before and after PSM.


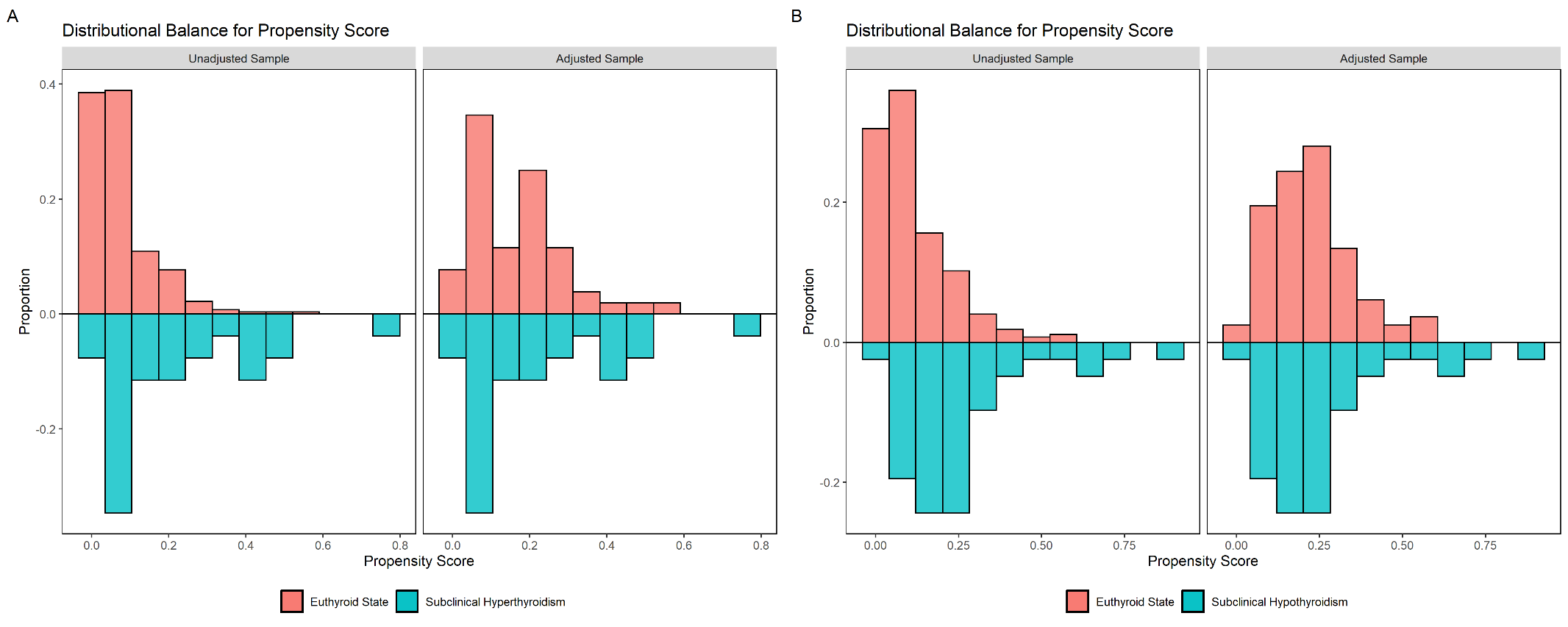


A: On the left, histograms of PS for the unadjusted populations who were with subclinical hyperthyroidism or euthyroid state. On the right, histograms of the propensity matched samples. B: On the left, histograms of PS for the unadjusted populations who were with subclinical hypothyroidism or euthyroid state. On the right, histograms of the propensity matched samples.

Figure S3 Standardized mean differences in the unmatched and matched sample


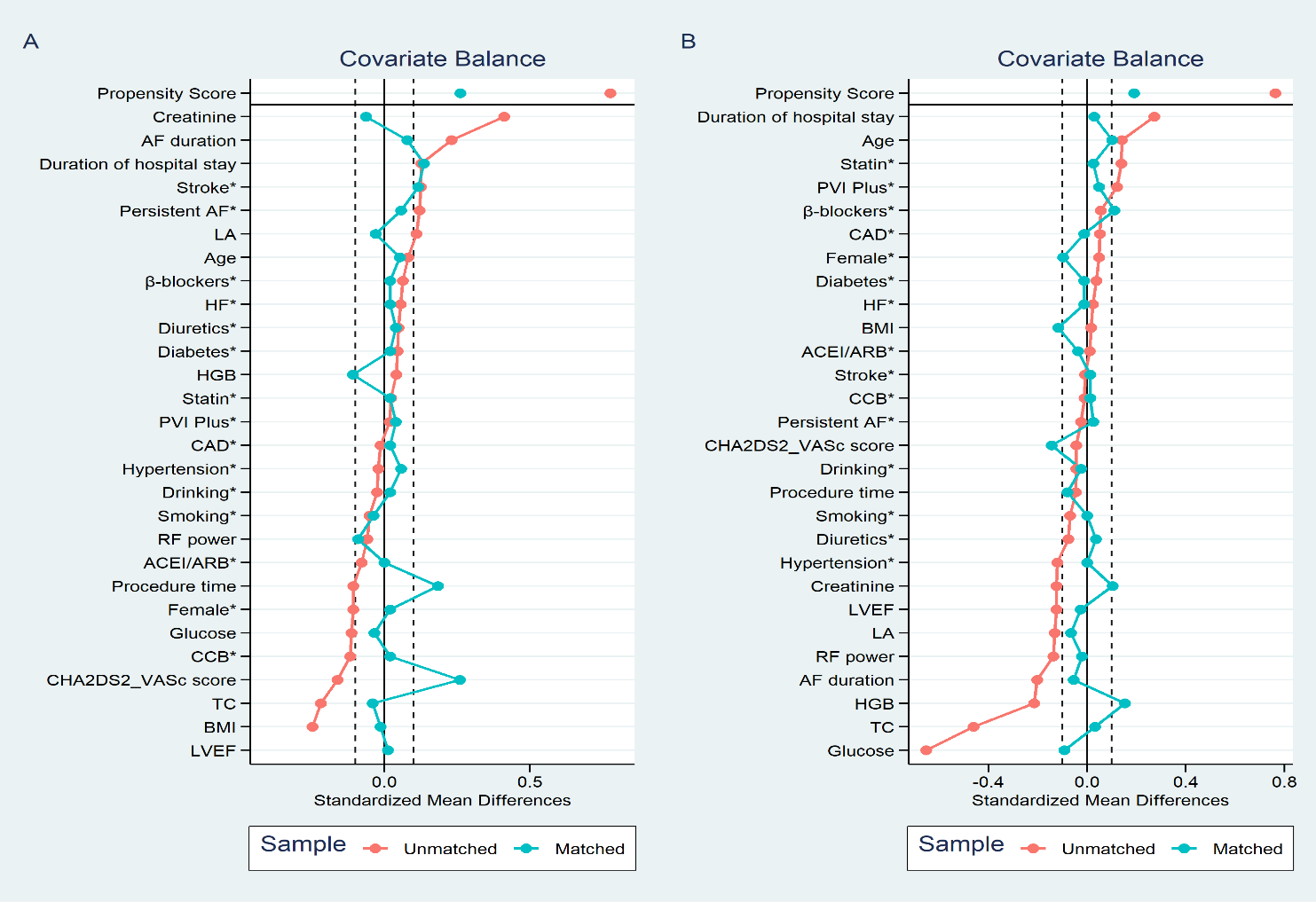


A: Standardized mean differences in the populations who were with subclinical hyperthyroidism or euthyroid state. B: Standardized mean differences in the populations who were with subclinical hypothyroidism or euthyroid state.

Figure S4 Distribution of inverse probability score weights in the subclinical hyperthyroidism, subclinical hypothyroidism and euthyroid state groups


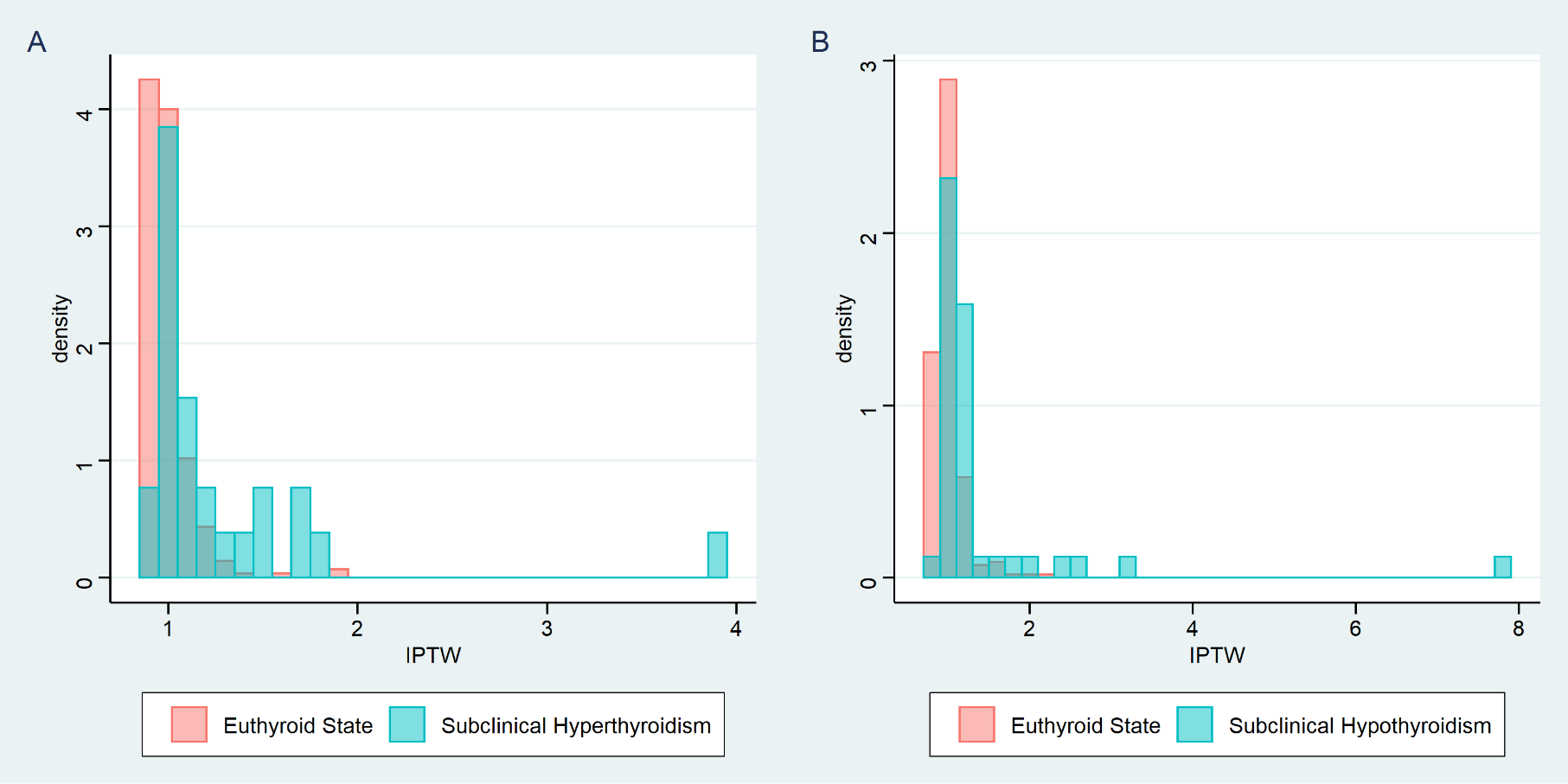


A: Inverse probability score weights in the populations who were with subclinical hyperthyroidism or euthyroid state. B: Inverse probability score weights in the populations who were with subclinical hypothyroidism or euthyroid state.

Table S1 Odds ratios (95% CIs) of patients with subclinical hyperthyroidism or subclinical hypothyroidism for all variables included in the propensity score models

|  | Subclinical Hyperthyroidism  VS Euthyroid state (n=301) | Subclinical hypothyroidism VS Euthyroid state (n=316) |
| --- | --- | --- |
| Age | 1.01 (0.96, 1.07) | 1.02 (0.97, 1.07) |
| BMI | 0.93 (0.78, 1.10) | 1.04 (0.92, 1.17) |
| Female | 1.31 (0.34, 5.11) | 0.59 (0.20, 1.75) |
| Smoking | 0.36 (0.06, 1.71) | 0.36 (0.07, 1.56) |
| Drinking | 1.24 (0.21, 5.98) | 1.15 (0.23, 4.94) |
| Persistent AF | 2.58 (0.88, 7.75) | 1.09 (0.42, 2.73) |
| AF duration | 1.01 (1.00, 1.01) | 1.00 (0.98, 1.00) |
| Duration of hospital stay | 1.09 (0.92, 1.28) | 1.14 (1.01, 1.29) |
| LAD | 0.96 (0.87, 1.04) | 0.92 (0.85, 0.99) |
| LVEF | 0.92 (0.87, 0.97) | 0.99 (0.93, 1.05) |
| HGB | 1.00 (0.97, 1.03) | 0.99 (0.96, 1.02) |
| Total cholesterol | 0.67 (0.38, 1.10) | 0.53 (0.31, 0.87) |
| Creatinine | 1.01 (0.99, 1.04) | 1.00 (0.97, 1.02) |
| Fasting glucose | 0.95 (0.65, 1.28) | 0.67 (0.42, 0.94) |
| CAD | 0.80 (0.18, 3.01) | 1.16 (0.42, 3.05) |
| Hypertension | 0.93 (0.30, 2.77) | 0.38 (0.14, 0.98) |
| Diabetes | 1.70 (0.37, 6.62) | 2.04 (0.59, 6.37) |
| Stroke | 6.68 (1.42, 33.68) | 0.75 (0.15, 3.22) |
| HF | 0.29 (0.04, 1.71) | 2.23 (0.38, 12.65) |
| Diuretics | 0.97 (0.25, 3.45) | 0.36 (0.10, 1.08) |
| Statin | 1.17 (0.39, 3.35) | 2.20 (1.00, 4.89) |
| ACEI/ARB | 1.07 (0.23, 4.46) | 1.51 (0.50, 4.45) |
| β-blockers | 1.44 (0.49, 4.14) | 1.33 (0.56, 3.07) |
| CCB | 0.26 (0.01, 1.95) | 0.93 (0.24, 3.12) |
| Procedure time | 0.99 (0.98, 1.01) | 1.00 (0.98, 1.01) |
| CHA2DS2VASc-score | 0.68 (0.40, 1.11) | 0.91 (0.61, 1.33) |
| PVI PLUS | 1.04 (0.18, 4.43) | 3.74 (1.17, 11.78) |
| RF power | 0.95 (0.87, 1.04) | 0.95 (0.89, 1.02) |

AF indicates atrial fibrillation; LAD, left atrium diameter; LVEF, left ventricular ejection fraction; BMI, body mass index; HGB, hemoglobin; CAD, coronary artery disease; HF, heart failure; ACEI, angiotensin-converting enzyme inhibitors; ARB, angiotensin-receptor blocker; CCB, calcium channel blocker; PVI Plus, cavotricuspid isthmus, superior vena cava, arrhythmogenic substrate modification or LA linear ablation beyond pulmonary vein isolation; RF power, radiofrequency power.

Table S2 Baseline characteristics of the participants after PSM

| Characteristic | Euthyroid  State  (n=52) | Subclinical Hyperthyroidism  (n=26) | *P* | Euthyroid  State  (n=82) | Subclinical Hypothyroidism  (n=41) | *P* |
| --- | --- | --- | --- | --- | --- | --- |
| Age(year) | 63.00 (57.00, 67.25) | 65.50 (55.50, 71.25) | 0.706 | 64.00 (56.50, 69.00) | 63.00 (57.00, 68.00) | 0.964 |
| BMI (kg/m2) | 24.11 (22.79, 26.15) | 23.94 (23.00, 25.30) | 0.849 | 25.47 (23.44, 27.49) | 24.44 (22.72, 27.68) | 0.350 |
| Gender (Female) | 15 (28.8) | 8 (30.8) | 1.000 | (46 (56.1) | 19 (46.3) | 0.406 |
| Current Smoking | 8 (15.4) | 3 (11.5) | 0.908 | 8 (9.8) | 4 (9.8) | 1.000 |
| Current Drinking | 5 (9.6) | 3 (11.5) | 1.000 | 10 (12.2) | 4 (9.8) | 0.920 |
| Persistent AF | 21 (40.4) | 12 (46.2) | 0.808 | 24 (29.3) | 13 (31.7) | 0.945 |
| CHA2DS2-VASc score | 1.00 (1.00, 2.00) | 1.50 (1.00, 3.00) | 0.383 | 2.00 (1.00, 3.00) | 2.00 (1.00, 3.00) | 0.677 |
| AF duration (months） | 31.79 (1.16, 60.99) | 24.35 (1.50, 72.98) | 0.787 | 11.57 (1.00, 36.42) | 5.87 (0.90, 24.33) | 0.509 |
| LAD (mm) | 38.00 (35.00, 42.00) | 37.50 (34.00, 45.00) | 0.886 | 37.00 (33.00, 41.00) | 36.00 (31.00, 41.00) | 0.632 |
| LVEF (%) | 61.00 (53.77, 66.10) | 61.20 (55.90, 66.53) | 0.903 | 65.85 (59.88, 69.30) | 65.00 (62.00, 66.70) | 0.506 |
| HGB(g/L) | 141.50 (132.75, 151.50) | 140.00 (127.75, 149.00) | 0.535 | 134.00 (126.00, 146.00) | 138.00 (130.00, 148.00) | 0.323 |
| Fasting glucose(mmol/L) | 5.11 (4.76, 5.72) | 5.11 (4.47, 6.19) | 0.759 | 4.97 (4.63, 5.48) | 4.93 (4.55, 5.63) | 0.780 |
| Creatinine(umol/L) | 79.00 (65.00, 86.25) | 80.00 (75.25, 85.25) | 0.574 | 65.85 (56.00, 80.50) | 71.00 (61.00, 80.00) | 0.302 |
| Total cholesterol(mmol/L) | 3.96 (3.42, 4.42) | 3.75 (3.05, 4.34) | 0.408 | 3.75 (3.25, 4.32) | 3.90 (3.23, 4.24) | 0.830 |
| CAD | 7 (13.5) | 4 (15.4) | 1.000 | 19 (23.2) | 9 (22.0) | 1.000 |
| Hypertension | 25 (48.1) | 14 (53.8) | 0.810 | 36 (43.9) | 18 (43.9) | 1.000 |
| Diabetes | 7 (13.5) | 4 (15.4) | 1.000 | 13 (15.9) | 6 (14.6) | 1.000 |
| Stroke | 6 (11.5) | 6 (23.1) | 0.318 | 7 (8.5) | 4 (9.8) | 1.000 |
| HF | 7 (13.5) | 4 (15.4) | 1.000 | 11 (13.4) | 5 (12.2) | 1.000 |
| Diuretics | 12 (23.1) | 7 (26.9) | 0.926 | 9 (11.0) | 6 (14.6) | 0.770 |
| Statin | 17 (32.7) | 9 (34.6) | 1.000 | 36 (43.9) | 19 (46.3) | 0.949 |
| ACEI/ARB | 8 (15.4) | 4 (15.4) | 1.000 | 23 (28.0) | 10 (24.4) | 0.829 |
| β-blockers | 21 (40.4) | 11 (42.3) | 1.000 | 25 (30.5) | 17 (41.5) | 0.313 |
| CCB | 1 (1.9) | 1 (3.8) | 1.000 | 11 (13.4) | 6 (14.6) | 1.000 |
| Procedure time (min) | 120.00 (99.50, 129.00) | 120.00 (108.75, 129.00) | 0.251 | 120.00 (101.75, 130.00) | 120.00 (90.00, 132.00) | 0.376 |
| RF power (W) | 37.50 (35.00, 40.00) | 35.00 (31.25, 40.00) | 0.685 | 35.00 (30.00, 40.00) | 35.00 (30.00, 40.00) | 0.905 |
| Duration of hospital stay (Days) | 7.00 (6.00, 9.00) | 7.00 (6.00, 8.00) | 0.699 | 8.00 (6.00, 10.00) | 8.00 (6.00, 10.00) | 0.662 |
| Ablation strategy (PVI Plus) | 4 (7.7) | 3 (11.5) | 0.889 | 14 (17.1) | 9 (22.0) | 0.683 |

AF indicates atrial fibrillation; LAD, left atrium diameter; LVEF, left ventricular ejection fraction; BMI, body mass index; HGB, hemoglobin; CAD, coronary artery disease; HF, heart failure; ACEI, angiotensin-converting enzyme inhibitors; ARB, angiotensin-receptor blocker; CCB, calcium channel blocker; PVI Plus, cavotricuspid isthmus, superior vena cava, arrhythmogenic substrate modification or LA linear ablation beyond pulmonary vein isolation; RF power, radiofrequency power; PSM, propensity score matching.

Data are given as medians (interquartile range) or frequencies (percentages), and compared using Mann-Whitney U test for continuous variables and the chi-squared test or Fisher’s exact test for categorical variables.

Table S3 Baseline characteristics of the participants after IPTW

| Characteristic | Euthyroid  State  (n=274) | Subclinical Hyperthyroidism  (n=33) | *P* | Euthyroid State  (n=275) | Subclinical Hypothyroidism  (n=58) | *P* |
| --- | --- | --- | --- | --- | --- | --- |
| Age(year) | 63.00 (56.00, 69.00) | 62.43 (50.00, 69.77) | 0.939 | 63.00 (56.00, 69.00) | 66.00 (57.00, 67.00) | 0.485 |
| BMI (kg/m2) | 24.77 (23.33, 27.04) | 23.58 (22.99, 24.80) | 0.130 | 24.82 (23.44, 27.06) | 23.85 (22.36, 27.01) | 0.346 |
| Gender (Female) | 110.9 (40.5) | 9.3 (27.8) | 0.217 | 115.9 (42.2) | 29.4 (50.8) | 0.408 |
| Current Smoking | 45.0 (16.4) | 3.0 (8.9) | 0.269 | 43.3 (15.8) | 4.9 (8.4) | 0.219 |
| Current Drinking | 38.6 (14.1) | 3.0 (9.0) | 0.435 | 38.0 (13.8) | 4.0 (6.8) | 0.162 |
| Persistent AF | 95.5 (34.8) | 17.6 (52.6) | 0.096 | 92.6 (33.7) | 17.9 (30.9) | 0.759 |
| CHA2DS2-VASc score | 2.00 (1.00, 3.00) | 1.10 (1.00, 3.00) | 0.585 | 2.00 (1.00, 3.00) | 2.00 (1.00, 3.00) | 0.647 |
| AF duration (months） | 12.23 (1.14, 46.84) | 24.37 (1.01, 108.79) | 0.215 | 12.19 (1.07, 37.37) | 1.20 (0.87, 24.18) | 0.012 |
| LAD (mm) | 38.00 (33.00, 41.00) | 37.99 (33.44, 45.00) | 0.451 | 38.00 (33.00, 41.00) | 34.91 (30.00, 40.00) | 0.156 |
| LVEF (%) | 66.10 (60.32, 70.12) | 59.05 (45.08, 65.43) | 0.003 | 66.11 (60.54, 70.20) | 64.70 (62.00, 66.70) | 0.021 |
| HGB(g/L) | 140.73 (131.00, 149.00) | 140.52 (127.00, 149.00) | 0.984 | 140.00 (130.00, 149.00) | 135.00 (127.00, 146.00) | 0.258 |
| Fasting glucose(mmol/L) | 5.20 (4.79, 5.78) | 5.12 (4.55, 6.09) | 0.834 | 5.18 (4.77, 5.73) | 4.87 (4.34, 5.29) | 0.018 |
| Creatinine(umol/L) | 72.00 (61.00, 83.00) | 80.45 (75.38, 88.16) | 0.003 | 71.00 (60.00, 82.60) | 70.00 (60.00, 79.83) | 0.528 |
| Total cholesterol(mmol/L) | 4.02 (3.53, 4.60) | 3.50 (2.90, 4.27) | 0.061 | 3.99 (3.51, 4.59) | 3.55 (3.03, 4.21) | 0.011 |
| CAD | 46.0 (16.8) | 4.6 (13.6) | 0.675 | 47.4 (17.3) | 17.1 (29.5) | 0.212 |
| Hypertension | 153.1 (55.9) | 19.4 (58.0) | 0.843 | 149.2 (54.4) | 23.4 (40.5) | 0.164 |
| Diabetes | 31.2 (11.4) | 5.3 (15.8) | 0.523 | 30.8 (11.2) | 8.8 (15.1) | 0.519 |
| Stroke | 31.6 (11.5) | 8.6 (25.8) | 0.055 | 28.8 (10.5) | 6.1 (10.4) | 0.994 |
| HF | 27.6 (10.1) | 7.4 (22.1) | 0.151 | 28.4 (10.4) | 5.3 (9.1) | 0.792 |
| Diuretics | 60.8 (22.2) | 11.6 (34.6) | 0.229 | 58.3 (21.2) | 7.4 (12.7) | 0.237 |
| Statin | 88.8 (32.4) | 13.4 (39.9) | 0.494 | 94.0 (34.2) | 32.0 (55.2) | 0.032 |
| ACEI/ARB | 61.9 (22.6) | 4.5 (13.5) | 0.273 | 63.5 (23.1) | 15.0 (25.9) | 0.742 |
| β-blockers | 101.1 (36.9) | 12.9 (38.4) | 0.881 | 99.6 (36.3) | 31.3 (54.0) | 0.072 |
| CCB | 40.3 (14.7) | 0.9 (2.7) | 0.049 | 42.5 (15.5) | 9.7 (16.8) | 0.859 |
| Procedure time (min) | 120.00 (100.00, 130.00) | 120.00 (89.51, 127.07) | 0.852 | 120.00 (100.00, 130.00) | 110.76 (98.01, 127.02) | 0.587 |
| RF power (W) | 35.00 (30.00, 40.00) | 35.00 (30.00, 40.62) | 0.891 | 35.00 (30.00, 40.00) | 35.00 (30.00, 40.00) | 0.974 |
| Duration of hospital stay (Days) | 7.00 (6.00, 9.00) | 7.00 (6.00, 10.82) | 0.541 | 7.00 (6.00, 9.00) | 9.00 (6.18, 10.00) | 0.007 |
| Ablation strategy (PVI Plus) | 27.4 (10.0) | 3.9 (11.6) | 0.807 | 29.9 (10.9) | 22.5 (38.8) | 0.001 |

AF indicates atrial fibrillation; LAD, left atrium diameter; LVEF, left ventricular ejection fraction; BMI, body mass index; HGB, hemoglobin; CAD, coronary artery disease; HF, heart failure; ACEI, angiotensin-converting enzyme inhibitors; ARB, angiotensin-receptor blocker; CCB, calcium channel blocker; PVI Plus, cavotricuspid isthmus, superior vena cava, arrhythmogenic substrate modification or LA linear ablation beyond pulmonary vein isolation; RF power, radiofrequency power; IPTW, inverse probability treatment weighting.

Data are given as medians (interquartile range) or frequencies (percentages), and compared using Mann-Whitney U test for continuous variables and the chi-squared test or Fisher’s exact test for categorical variables.

Table S4 Recurrence of atrial fibrillation by fT3, fT4, TSH quintiles and fT3, fT4, TSH as a continuous exposure based on the non-transformed data.

| **Variable** | **fT3(pmol/L)** | | | | **fT4(pmol/L)** | | | | **TSH(mIU/L)** | | | |
| --- | --- | --- | --- | --- | --- | --- | --- | --- | --- | --- | --- | --- |
|  | **IQR(pmol/L)** | **Events/N** | **Crude Model**  **(HR,95%CI)** | **Adjusted Model**  **(HR,95%CI)** | **IQR(pmol/L)** | **Events/N** | **Crude Model**  **(HR,95%CI)** | **Adjusted Model**  **(HR,95%CI)** | **IQR(mIU/L)** | **Events/N** | **Crude Model**  **(HR,95%CI)** | **Adjusted Model**  **(HR,95%CI)** |
| Per 1 unit increase | -- | 91/342 | 1.12  (0.94,1.34) | 1.15 (0.91, 1.45) | -- | 91/342 | 0.93 (0.86, 1.02) | 0.93 (0.85, 1.03) | -- | 91/342 | 0.92 (0.82, 1.02) | 0.89 (0.79, 0.99) |
| Quintiles |  |  |  |  |  |  |  |  |  |  |  |  |
| Q1 | (3.59-4.19) | 14/69 | Ref | Ref | (12.30-13.53) | 24/69 | Ref | Ref | (0.33-0.83) | 20/69 | Ref | Ref |
| Q2 | (4.43-4.60) | 19/69 | 1.44  (0.72,2.87) | 1.80 (0.84, 3.85) | (14.22-14.90) | 18/68 | 0.75  (0.41, 1.39) | 0.57 (0.29, 1.12) | (1.16-1.44) | 19/69 | 1.00  (0.53, 1.87) | 1.06 (0.54, 2.11) |
| Q3 | (4.78-4.96) | 14/68 | 1.04  (0.49, 2.18) | 0.85 (0.38, 1.88) | (15.40-16.00) | 13/68 | 0.55  (0.28, 1.08) | 0.58 (0.28, 1.18) | (1.71-2.14) | 18/67 | 0.97  (0.51, 1.83) | 0.88 (0.44, 1.76) |
| Q4 | (5.20-5.44) | 18/67 | 1.43  (0.71, 2.88) | 1.91 (0.90, 4.05) | (16.59-17.38) | 20/68 | 0.90  (0.50, 1.63) | 0.90 (0.47, 1.76) | (2.44-3.00) | 19/68 | 1.08  (0.58, 2.03) | 0.94 (0.47, 1.89) |
| Q5 | (5.71-6.44) | 26/69 | 2.23  (1.16, 4.28) | 2.13 (1.04, 4.37) | (18.30-21.20) | 16/69 | 0.66  (0.35, 1.25) | 0.62 (0.32, 1.22) | (3.91-6.59) | 15/69 | 0.73  (0.37, 1.43) | 0.70 (0.34, 1.41) |
| *P* for trend | -- | -- | 0.024 | 0.055 | -- | -- | 0.342 | 0.463 | -- | -- | 0.485 | 0.291 |

TSH indicates thyroid stimulating hormone; fT4, free thyroxine; fT3, free triiodothyronine; AF, atrial fibrillation; CI, confidence interval; HR, hazard ratio; SD, standard deviation; IQR, interquartile range.

Adjusted Model: adjust for age, BMI, gender, current smoking and drinking status, AF pattern, CHA2DS2-VASc score, AF duration, echocardiogram information, past diagnoses, current medications, laboratory tests and procedure parameters.

Table S5 Hazard ratios (95% CIs) for the endpoint for all variables included as covariates in the Univariable and Multivariable Cox model

|  | Univariable model | Model1 | model2 | model3 |
| --- | --- | --- | --- | --- |
| Subclinical Hyperthyroidism | 1.98(1.10-3.59) | 2.12 (1.17, 3.86) | 2.93 (1.56, 5.53) | 3.07 (1.54, 6.14) |
| Subclinical Hypothyroidism | 0.81(0.40-1.61) | 0.80 (0.40, 1.60) | 0.71 (0.34, 1.46) | 0.66 (0.31, 1.43) |
| Age | 0.98(0.96-1.00) | 0.98 (0.96, 1.00) | 0.95 (0.93, 0.98) | 0.95 (0.92, 0.97) |
| BMI | 1.04(0.98-1.11) |  |  | 1.03 (0.96, 1.10) |
| Female | 1.53(1.01-2.31) | 1.73 (1.14, 2.64) |  | 1.26 (0.65, 2.44) |
| Smoking | 0.54(0.27-1.08) |  | 0.58 (0.28, 1.18) | 0.62 (0.26, 1.44) |
| Drinking | 0.73(0.37-1.45) |  |  | 1.13 (0.49, 2.58) |
| Persistent AF | 1.53(1.01-2.32) |  |  | 1.36 (0.81, 2.28) |
| AF duration | 1.00(1.00-1.01) |  |  | 1.00 (1.00, 1.01) |
| Duration of hospital stay | 0.93(0.86-1.01) |  | 0.91 (0.84, 0.99) | 0.90 (0.82, 0.98) |
| LAD | 1.08(1.04-1.12) |  | 1.09 (1.05, 1.13) | 1.08 (1.04, 1.12) |
| LVEF | 1.00(0.98-1.02) |  |  | 1.01 (0.98, 1.03) |
| HGB | 0.99(0.97-1.00) |  | 0.97 (0.95, 0.98) | 0.97 (0.95, 0.99) |
| Total cholesterol | 0.96(0.76-1.21) |  |  | 1.07 (0.84, 1.36) |
| Creatinine | 1.00(0.99-1.01) |  |  | 1.00 (0.99, 1.02) |
| Fasting glucose | 1.03(0.90-1.19) |  |  | 1.07 (0.92, 1.25) |
| CAD | 1.33(0.78-2.25) |  |  | 1.64 (0.87, 3.12) |
| Hypertension | 0.69(0.46-1.04) |  | 0.51 (0.30, 0.87) | 0.48 (0.27, 0.85) |
| Diabetes | 1.14(0.61-2.14) |  |  | 0.90 (0.45, 1.81) |
| Stroke | 0.82(0.41-1.63) |  | 0.40 (0.17, 0.94) | 0.43 (0.17, 1.09) |
| HF | 1.65(0.92-2.97) |  | 1.82 (0.88, 3.74) | 1.98 (0.78, 5.04) |
| Diuretics | 0.89(0.54-1.48) |  | 0.56 (0.30, 1.06) | 0.58 (0.29, 1.14) |
| Statin | 0.86(0.55-1.35) |  |  | 0.80 (0.48, 1.32) |
| ACEI/ARB | 1.34(0.85-2.13) |  | 2.22 (1.26, 3.91) | 2.24 (1.24, 4.05) |
| β-blockers | 1.02(0.67-1.56) |  |  | 0.85 (0.52, 1.40) |
| CCB | 0.86(0.48-1.55) |  |  | 0.94 (0.46, 1.95) |
| Procedure time | 1.00(1.00-1.01) |  | 1.01 (1.00, 1.01) | 1.00 (1.00, 1.01) |
| CHA2DS2VASc-score | 1.01(0.88-1.16) |  | 1.33 (1.09, 1.63) | 1.33 (1.04, 1.71) |
| PVI PLUS | 1.48(0.83-2.67) |  |  | 1.45 (0.76, 2.79) |
| RF power | 1.03(0.99-1.07) |  | 1.06 (1.02, 1.11) | 1.05 (1.01, 1.10) |

AF indicates atrial fibrillation; LAD, left atrium diameter; LVEF, left ventricular ejection fraction; BMI, body mass index; HGB, hemoglobin; CAD, coronary artery disease; HF, heart failure; ACEI, angiotensin-converting enzyme inhibitors; ARB, angiotensin-receptor blocker; CCB, calcium channel blocker; PVI Plus, cavotricuspid isthmus, superior vena cava, arrhythmogenic substrate modification or LA linear ablation beyond pulmonary vein isolation; RF power, radiofrequency power.

The results in this table are provided for the reader’s information but should not be interpreted to provide information on predictors or causes of the outcome.

Table S6 Associations between subclinical thyroid dysfunction and the recurrence rate after radiofrequency catheter ablation for atrial fibrillation in the Crude, Multivariate and PS Analyses based on the other imputed datasets.

| Analyses | Dateset2 | Dateset3 | Dateset4 | Dateset5 |
| --- | --- | --- | --- | --- |
| No. of events / No. at risk (%) |  |  |  |  |
| Subclinical Hyperthyroidism | 13/26(50.0) | 13/26(50.0) | 13/26(50.0) | 13/26(50.0) |
| Euthyroid State | 69/275(25.1) | 69/275(25.1) | 69/275(25.1) | 69/275(25.1) |
| Subclinical Hypothyroidism | 9/41(22.0) | 9/41(22.0) | 9/41(22.0) | 9/41(22.0) |
| Crude analyses— HR (95% CI)  Ref= Euthyroid State |  |  |  |  |
| Subclinical Hyperthyroidism | 1.98 (1.10-3.59) | 1.98 (1.10-3.59) | 1.98 (1.10-3.59) | 1.98 (1.10-3.59) |
| Subclinical Hypothyroidism | 0.81 (0.40-1.61) | 0.81 (0.40-1.61) | 0.81 (0.40-1.61) | 0.81 (0.40-1.61) |
| Multivariable analyses— HR (95% CI)  Ref= Euthyroid State* |  |  |  |  |
| Model1 |  |  |  |  |
| Subclinical Hyperthyroidism | 2.12 (1.17, 3.86) | 2.12 (1.17, 3.86) | 2.12 (1.17, 3.86) | 2.12 (1.17, 3.86) |
| Subclinical Hypothyroidism | 0.80 (0.40, 1.60) | 0.80 (0.40, 1.60) | 0.80 (0.40, 1.60) | 0.80 (0.40, 1.60) |
| Model2 |  |  |  |  |
| Subclinical Hyperthyroidism | 2.13 (1.13, 4.00) | 2.17 (1.16, 4.07) | 2.09 (1.10, 3.99) | 2.62 (1.41, 4.88) |
| Subclinical Hypothyroidism | 0.72 (0.36, 1.47) | 0.63 (0.30, 1.31) | 0.59 (0.29, 1.21) | 0.82 (0.40, 1.71) |
| Model3 |  |  |  |  |
| Subclinical Hyperthyroidism | 2.52 (1.27, 4.98) | 2.30 (1.18, 4.49) | 2.16 (1.09, 4.29) | 2.69 (1.38, 5.27) |
| Subclinical Hypothyroidism | 0.69 (0.33, 1.47) | 0.58 (0.27, 1.23) | 0.57 (0.27, 1.22) | 0.74 (0.34, 1.60) |
| PS analyses— hazard ratio (95% CI)  Ref= Euthyroid State |  |  |  |  |
| With IPTW† |  |  |  |  |
| Subclinical Hyperthyroidism | 2.47 (1.28, 4.77) | 2.40 (1.28, 4.51) | 1.97 (1.00, 3.89) | 2.58 (1.38, 4.83) |
| Subclinical Hypothyroidism | 0.61 (0.31, 1.21) | 0.54 (0.26, 1.11) | 0.48 (0.22, 1.02) | 0.54 (0.24, 1.23) |
| With PSM‡ |  |  |  |  |
| Subclinical Hyperthyroidism | 2.45 (1.10, 5.48) | 1.96 (0.91, 4.23) | 2.07 (0.94, 4.56) | 2.46 (1.10, 5.51) |
| Subclinical Hypothyroidism | 0.59 (0.28, 1.24) | 0.77 (0.36, 1.67) | 0.72 (0.33, 1.56) | 0.86 (0.39, 1.88) |
| Adjusted for PS§ |  |  |  |  |
| Subclinical Hyperthyroidism | 2.81 (1.41, 5.62) | 2.79 (1.40, 5.58) | 2.89 (1.43, 5.84) | 3.38 (1.68, 6.77) |
| Subclinical Hypothyroidism | 0.63 (0.29, 1.37) | 0.58 (0.27, 1.25) | 0.52 (0.24, 1.14) | 0.68 (0.31, 1.49) |

*Model1 with additional adjustment for age, gender; model2 with additional adjustment for age, current smoking status, duration of hospital stay, LAD, HGB, hypertension, stroke, HF, diuretics, ACEI/ARB, procedure time, RF power using Akaike Information Criterion (AIC) for model selection. Model3 with additional adjustment for age, BMI, gender, current smoking and drinking status, AF pattern, CHA2DS2-VASc score, AF duration, echocardiogram information, past diagnoses, current medications, laboratory tests and procedure parameters. The analysis includes all 342 patients.

†Adjust for the same covariates in model3 with IPTW according to the PS. The analysis includes all 342 patients.

‡Without additional adjustment because of the well balance of the covariates after PSM. The analysis includes 78 patients (26 in subclinical hyperthyroidism state and 52 in euthyroid state) and 123 patients (41 in subclinical hypothyroidism state and 82 in euthyroid state), respectively.

§Adjust for the same covariates in model3, with additional adjustment for the PS. The analysis includes all 342 patients.

HR indicates hazard ratio, CI indicates confidence interval, PSM indicates propensity score matching, PS indicates propensity score, IPTW denotes inverse probability treatment weighting.
